# YAP1/TAZ drives ependymoma-like tumour formation in mice

**DOI:** 10.1038/s41467-020-16167-y

**Published:** 2020-05-13

**Authors:** Noreen Eder, Federico Roncaroli, Marie-Charlotte Dolmart, Stuart Horswell, Felipe Andreiuolo, Helen R. Flynn, Andre T. Lopes, Suzanne Claxton, John-Paul Kilday, Lucy Collinson, Jun-Hao Mao, Torsten Pietsch, Barry Thompson, Ambrosius P. Snijders, Sila K. Ultanir

**Affiliations:** 1 Kinases and Brain Development Laboratory, The Francis Crick Institute, London, NW1 1AT UK; 2 Protein Analysis and Proteomics Platform, The Francis Crick Institute, London, NW1 1AT UK; 3 Manchester Centre for Clinical Neuroscience, Salford Royal NHS Foundation Trust, Salford and Division of Neuroscience and Experimental Psychology, Faculty of Biology, Medicine and Health, School of Biology, University of Manchester, Manchester, M13 9PT UK; 4 Electron Microscopy Platform, The Francis Crick Institute, London, NW1 1AT UK; 5 Bioinformatics and Biostatistics Platform, The Francis Crick Institute, London, NW1 1AT UK; 6 Institute of Neuropathology, DGNN Brain Tumour Reference Center, University of Bonn, Bonn, Germany; 7 Centre for Paediatric, Teenage and Young Adult Cancer, Faculty of Biology, Medicine and Health, University of Manchester, Manchester, M13 9PT UK; 8 Department of Molecular, Cell and Cancer Biology, University of Massachusetts Medical School, Worcester, MA 01605 USA; 9 Epithelial Biology Laboratory, The Francis Crick Institute, London, NW1 1AT UK

**Keywords:** Cancer models, CNS cancer, Tumour-suppressor proteins, Phosphorylation, Cell proliferation

## Abstract

YAP1 gene fusions have been observed in a subset of paediatric ependymomas. Here we show that, ectopic expression of active nuclear YAP1 (nlsYAP5SA) in ventricular zone neural progenitor cells using conditionally-induced NEX/NeuroD6-Cre is sufficient to drive brain tumour formation in mice. Neuronal differentiation is inhibited in the hippocampus. Deletion of YAP1’s negative regulators LATS1 and LATS2 kinases in NEX-Cre lineage in double conditional knockout mice also generates similar tumours, which are rescued by deletion of YAP1 and its paralog TAZ. YAP1/TAZ-induced mouse tumours display molecular and ultrastructural characteristics of human ependymoma. RNA sequencing and quantitative proteomics of mouse tumours demonstrate similarities to YAP1-fusion induced supratentorial ependymoma. Finally, we find that transcriptional cofactor HOPX is upregulated in mouse models and in human YAP1-fusion induced ependymoma, supporting their similarity. Our results show that uncontrolled YAP1/TAZ activity in neuronal precursor cells leads to ependymoma-like tumours in mice.

## Abstract (web summary)

YAP1 gene fusions are found in subgroups of paediatric ependymomas. Here the authors show that YAP1 activation in NeuroD6 positive neuronal precursor cells can induce ependymoma-like tumours in mice.

## Introduction

Yes-associated protein 1 (YAP1) is a transcriptional regulator with oncogenic activity that is involved in cellular proliferation, maintenance of stem cell properties and tumorigenesis^1^. YAP1 activity is the output of the conserved hippo signalling pathway, which is implicated in cancer^2–4^. This canonical kinase signalling system/pathway transmits mechanical stimuli from the cell surface to regulate cell proliferation, survival and tissue size in multiple organs across species^1,5–8^. The core kinase cascade consists of the Hippo kinase in *Drosophila* and its homologue Mammalian Sterile 20-like 1 (MST1) and MST2 in mammals, together with hippo’s downstream effector Warts in *Drosophila*, which is homologous to the Large tumour suppressor homologue 1 (LATS1) and LATS2 kinases in mammals^9^. MST1/2 phosphorylates and activate LATS1/2, which in turn phosphorylates to inhibit YAP1 or its close homologue TAZ/WWTR1. YAP1 phosphorylation promotes its degradation via 14-3-3 binding and cytoplasmic sequestering, preventing its translocation to the nucleus^10^. YAP1 activates TEAD1-4-dependent gene expression, which is required for its effects on cellular proliferation^11–14^. The role of hippo signalling in mammalian tumorigenesis has been demonstrated in vivo, for example, by conditional deletion of MST1/2 or LATS1/2 in liver, which deregulates progenitor cell proliferation and leads to tumorigenesis in mice^15–17^. Moreover, activated YAP1 expression has been shown to drive embryonal rhabdomyosarcoma formation^18^, whereas its expression in several cancer types makes YAP1 a considered target for novel treatments^19^. However, its role in brain tumours, such as ependymoma, is not fully described.

Ependymoma is the third most common paediatric brain tumour. It accounts for 5–10% of all primary tumours of the central nervous system (CNS) in children and adolescents^20–23^. Ependymomas have recently been classified into nine molecular subgroups based on genetic and epigenetic profiles, age of onset and location (supratentorial, posterior fossa and spinal)^24,25^. Surgery followed by radiotherapy are the main treatment modalities for most patients^24,26–28^. Recurrence rates are however high and up to 40% of ependymomas are incurable^24,27^. Approximately 70% of supratentorial ependymomas are characterised by a fusion between C11ORF95 and RELA^29,30^, whereas ~4–10% carry the fusions of YAP1 with other genes encoding transcription factors: YAP1-MAMLD1, or YAP1-FAM118B^29,31,32^. The group of ependymomas with YAP1 fusions occurs almost exclusively in children^24^.

Ependymomas likely originate from a restricted population of radial glial-like stem cells that are found in the ventricles and spinal canal of embryos and adults^33^. Radial glia and intermediate neural progenitors derived from them normally produce neurons, oligodendrocytes, astrocytes and ependymal cells^34–38^. Signalling mechanisms that normally regulate cellular progenitor proliferation and orchestrate differentiation are therefore strong candidates to be involved in paediatric tumourigenesis. YAP1 is present in the embryonic ventricular zone and later in the ependyma of mice^39,40^ where it has been implicated in ependymal cell differentiation^40^.

We use conditional mouse models of active YAP1 expression and LATS1/2 deletion and show that suppression of YAP1 activity in NEX/NeuroD6 expressing neuronal precursor cells is essential for limiting proliferation and enabling neuronal differentiation in the brain, whereas activation of YAP1 is sufficient to produce tumours that display features similar to human ependymoma.

## Results

### Active YAP1 expression in NEX-Cre lineage induces tumours

**Fig. 1:**
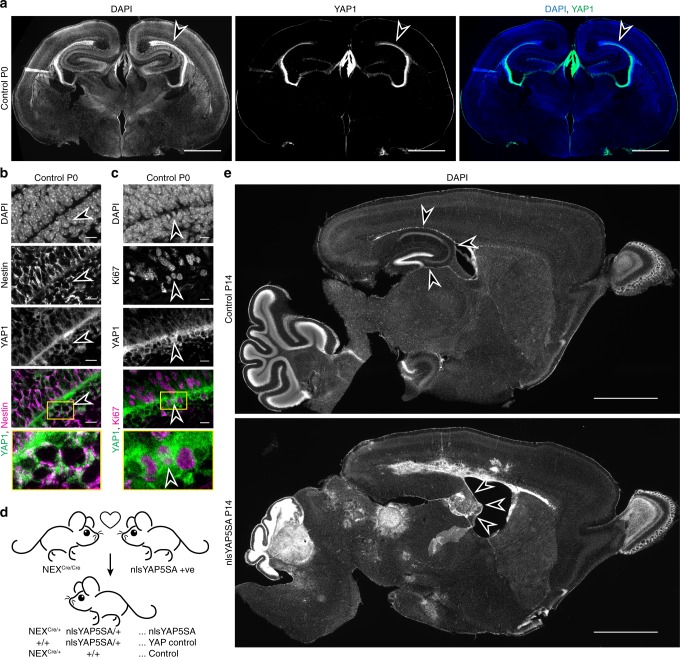
YAP1 is present in ependymal layer and its regulation is critical for brain development. **a** Coronal view of control P0 mouse brain sections immunofluorescence stained for YAP1. Arrowhead pointing at strong positive staining in the ependymal layer, scale bars = 1 mm. **b**–**c** Higher magnifications of ependymal layer/ventricular zone in control mice at P0. **b** Immunostainings with YAP1 and Nestin and **c**. Immunostainings with YAP1 and Ki67 show colocalization (arrows point to cells with colocalizations, overlap shown in white colour). Scale bars = 10 µm. **d** Breeding scheme to generate mice expressing nlsYAP5SA under the control of NEX-Cre (referred to as nlsYAP5SA). Mice with only one allele of NEX-Cre without the YAP1 transgene were used as controls. **e** Sagittal vibratome sections of the whole brain of Control and nlsYAP5SA mice are shown at P14. In nlsYAP5SA mice, ependymal layer is destructed, multiple tumours are formed across the brain as shown by DAPI stained nuclei and hippocampus is not developed. Arrowheads point at hippocampus. Scale bar = 1 mm.

Whether or not elevated YAP1 activity is tumourigenic in the developing mammalian brain is an open question. To target for cell types that normally express YAP1 in a developing brain, we first examined YAP1 localisation in mice brains using immunohistochemistry (IHC) with a specific anti-YAP1 antibody. YAP1 was highly expressed in the ventricular zone (VZ) at postnatal day 0 (P0) (Fig. 1a, Supplementary Fig. 1a), becoming localised in the ependymal layer at P20 (Supplementary Fig. 1a)^41^. A subset of YAP1-positive cells in the VZ at P0 was mitotically active and were of radial glial origin as shown by Ki67 and nestin co-expression, respectively (Fig. 1b, c). NEX-Cre positive neuronal precursor cells (NPCs) are known to be generated in the VZ starting at E11.5 and NEX/Math2/NeuroD6 lineage gives rise to pyramidal neurons of the cortex and hippocampus but does not contribute to astrocytes or oligodendrocytes^42,43^. We crossed NEX-Cre-expressing mice with a Rosa26-tdTomato mouse reporter line^44^. In addition to pyramidal neurons, we observed numerous tdTomato expressing cells in the VZ at P0 (Supplementary Fig. 1b–d). This finding is in agreement with previous reports showing NEX-Cre in dividing NPCs^42^. TdTomato reporter positive cells also expressed YAP1, indicating that NEX-Cre driver can be used to manipulate a sub-population of neural precursor cells (Supplementary Fig. 1b–d).

Based on this evidence, we assessed the effect of uncontrollable YAP1 activity in NEX-expressing NPCs, by using a Cre inducible mouse model, in which a hyperactive form of YAP1, nlsYAP5SA, is preceded by a floxed STOP codon^45^. Upon Cre-mediated recombination these transgenic mice express phosphomutant YAP1 (S61A, S109A, S127A, S164A, S381A), which cannot be phosphorylated and inhibited by kinases, such as LATS1/2^46^. In addition, nlsYAP5SA contains a nuclear localisation signal (nls) to direct YAP1 activity to the nucleus. nlsYAP5SA; NEX-Cre mice (Fig. 1d) displayed multiple subependymal tumours at P14 along the lateral ventricles as well as in cerebellum (Fig. 1e). These results indicate that uncontrolled YAP1 in NEX-Cre lineage is sufficient for tumour formation.

### nlsYAP5SA suppresses hippocampal neuron differentiation

**Fig. 2:**
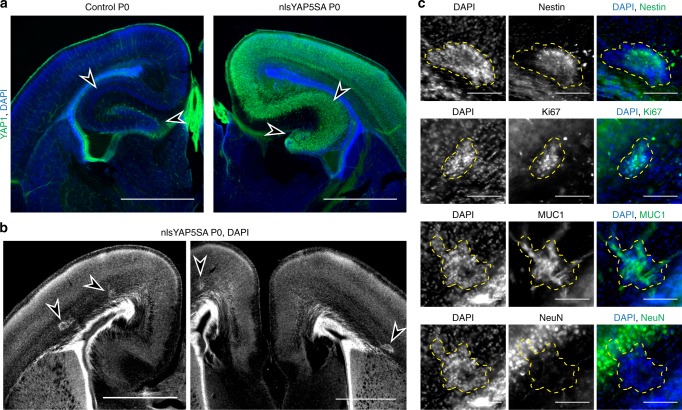
Uncontrolled YAP1 in NEX-Cre-expressing cells is sufficient for tumour formation at the expense of hippocampal differentiation. **a** Immunofluorescence staining of YAP1 in coronal vibratome sections of P0 animals. Neurons in nlsYAP5SA mouse cortex show strong nuclear staining of YAP1. Hippocampus formation is altered in nlsYAP5SA (arrowheads). Scale bars = 1 mm. **b** Arrowheads pointing at tumours visible in coronal vibratome sections of a P0 nlsYAP5SA animal. Scale bars = 1 mm. **c** Immunofluorescence staining in coronal sections of tumours in a nlsYAP5SA mice. Tumours are outlined with yellow-dashed line. Nestin, Ki67 and MUC1 demonstrate highly intense staining in the tumours while NeuN is absent. Scale bars = 100 µm.

We next determined the progression of the tumour formation at earlier developmental stages. In nlsYAP5SA; NEX-Cre mice, nuclear YAP1 was expressed throughout neocortex and hippocampus (Fig. 2a). In these animals, hippocampal neuronal cell body layers (CA1, CA3 and dentate gyrus) showed neuronal loss and disruption of normal layering as observed with the neuronal markers NeuN and CTIP2 (Fig. 2a, Supplementary Fig. 2a, b). We also identified multiple tumours adjacent to the ependymal layer (Fig. 2b). These tumours were positive for nestin, indicating that constitutive cells are of neuronal stem cell specification (Fig. 2c). Tumours did not express NeuN indicating that the tumour did not contain differentiated neurons, but several cells within the tumour mass expressed Ki67, a marker of proliferation, indicating that the tumours contain actively dividing cells (Fig. 2c). These data show that sustained nuclear YAP1 in NEX-Cre expressing NPCs lead to tumour formation by maintaining a neural stem cell-like fate and preventing hippocampal pyramidal neuronal differentiation.

### Deletion of LATS1/2 in NEX-Cre lineage causes brain tumours

**Fig. 3:**
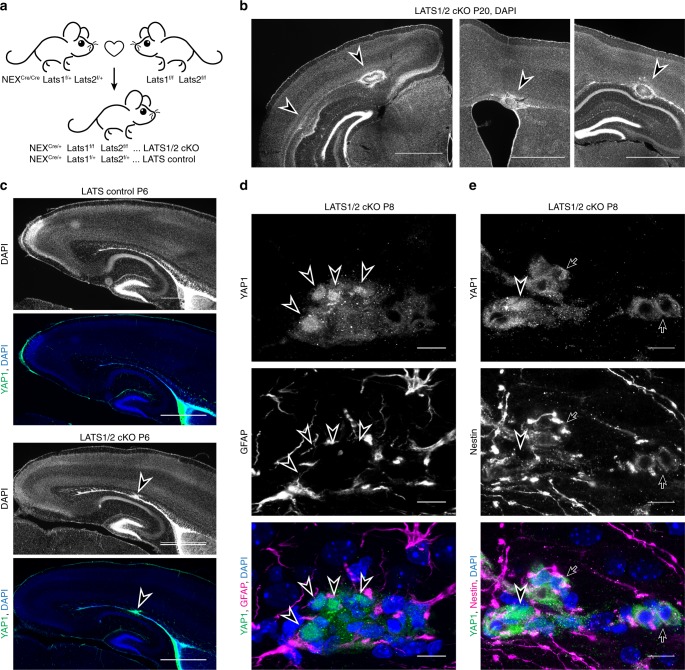
Conditional knockout of LATS1/2 under control of NEX-Cre leads to clonal YAP1-positive tumours. **a** Breeding scheme to generate LATS1/2 conditional knockout (LATS1/2 cKO) mice and control littermates. **b** Tumours associated with the ependymal layer are found in LATS1/2 cKO mice at P20 (arrowheads). Multiple clonal tumours can be found in each brain (middle and right images are from the same brain at different coronal planes). Scale bars = 1 mm. **c** A coronal section of LATS1/2 cKO brain at P6 shows strong YAP1 immunofluorescence staining in a tumour arising from the ependymal layer (arrowhead). Scale bars =  mm. **d**–**e** Confocal images of immunofluorescence stainings in LATS1/2 cKO brains. Scale bars = 10 µm. **d** YAP1 staining is increased in the tumour and YAP1 is nuclear in some cells (arrowheads). YAP1 expression does not overlay with GFAP^+^ cells. **e** YAP1 and Nestin are co-expressed in the tumour cells (arrows).

Despite several lines of evidence that supports an oncogenic role for YAP1, the impact of kinases regulating YAP1 activity in cancer is not well understood. In mammals, the canonical upstream phosphorylation and suppression of YAP1/TAZ activity is predominantly accomplished by two homologous kinases, LATS1 and LATS2. Constitutive knockout of LATS2 results in embryonic lethality^47^, whereas constitutive LATS1 knockout leads to soft tissue sarcoma and ovarian tumour development^48^, indicating essential roles of LATS2 during development and compensatory roles between the homologues. Conditional knockout of LATS1 and LATS2 (LATS1/2) in mouse liver increases proliferation and represses the maturation of hepatocytes thereby causing pre-natal lethality^17^. Whether or not LATS1/2 has a role in neuronal differentiation in mammals is not known. We tested if deficiency in YAP1 phosphoregulation by LATS1/2 is sufficient to cause tumour formation by conditionally knocking out Lats1 and Lats2, in NEX-Cre lineage (Fig. 3a). We found that dual LATS1/2 conditional knockouts (cKOs), but not individual LATS1 cKOs or LATS2 cKOs, have significantly reduced body weight by P19-P21 compared with control littermates, with an onset of difference at P15 (Supplementary Fig. 3a, b). Upon inspection of LATS1/2 cKO mice at P0–P21 age groups, we found that 67% at P5–P7 and 100% at P8–P21 developed tumours whereas no tumours were found before P5 (Supplementary Fig. 3c). Multiple clonal forebrain tumours, originating invariably at the ependymal layer were found in all LATS1/2 cKOs, but not in controls or individual LATS1 cKOs or LATS2 cKO at P20 (18/18 dual knockout, 0/3 LATS1 cKO, 0/3 LATS2 cKO, 0/6 controls), indicating redundancy between LATS1 and LATS2. Animals showed multifocal, discrete tumours indicating their synchronous origin (Fig. 3b). LATS1/2 cKO brains were collected at predetermined time points (mostly at P20) as permitted by our project licence and were not assessed by survival (see methods). Based on the severity indications, we believe the majority of LATS1/2 cKO mice and all nlsYAP5SA mice were moribund before 2 months of age. YAP1 was expressed in all tumours including the smallest lesions observed in younger animals. This finding indicates that loss of LATS1/2 function increases YAP1 activity (Fig. 3c). In LATS1/2 deletion induced tumours, YAP1 was highly expressed in the cytoplasm of all cells and was enriched in the nucleus in a subset of cells in tumour, in a mosaic fashion (Fig. 3d, e). Although some cells expressed high levels of YAP1 in the nucleus, some had less-intense YAP1 levels in nucleus (Fig. 3d, e). These results indicate that in LATS1/2 cKO tumours YAP1 nuclear localisation can be regulated by additional factors, such as actomyosin and Src kinase, as previously shown^49^. Furthermore, YAP1-positive tumours in this early stage of development showed increased nestin expression and nestin was present in cells expressing nuclear YAP1 as well as those that had increased YAP1 in cytoplasm (Fig. 3e). In addition to the periventricular tumours, we observed tumour formation in the cerebellum (Supplementary Fig. 3d), recapitulating the features of nlsYAP5SA; NEX-Cre mice (Fig. 1e). These data show that LATS1/2 loss in NEX-Cre expressing neural precursor cells cause YAP1-expressing tumours that are highly similar to tumours found in nlsYAP5SA; NEX-Cre mice. Our finding of much smaller clonal tumour formation in LATS1/2 cKO mice, when compared with nlsYAP5SA; NEX-Cre mice is likely owing to other regulators of YAP1/TAZ activity, such as Src and NDR kinases^50,51^. Therefore, LATS1/2 activity in NPCs is necessary to control YAP1 activity during development.

### Tumours in LATS1/2 conditional knockout mice are YAP1/TAZ dependent

**Fig. 4:**
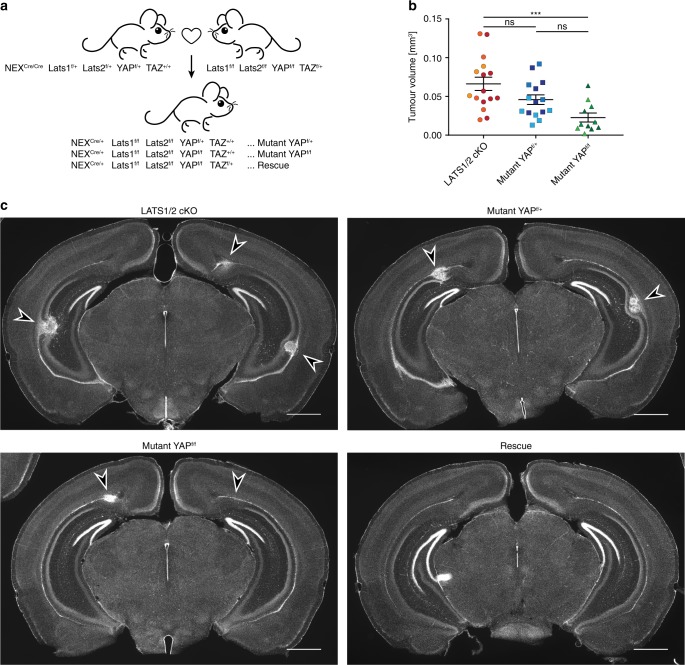
YAP1 and TAZ are necessary for tumour development in LATS1/2 cKO mice. **a** Breeding scheme to generate a rescue model. Available animals (Lats1^f/f^ Lats2^f/f^, YAP^f/f^ TAZ^f/+^ and NEX^Cre/Cre^) were crossed to generate mice with shown genotypes. **b** Measurements of size of tumours found in LATS1/2 cKO (*n* = 16 tumours), Mutant YAP^f/+^ (*n* = 15 tumours) and Mutant YAP^f/f^ (*n* = 11 tumours) from *n* = 3 mice for each genotype. Colour shade indicates tumours from the same animal. LATS1/2 cKO tumour volumes compared with those measured in Mutant YAP^f/f^ animals show a significant difference with padj = 0.0006 (two-sided Dunn’s multiple comparison test). Error bars = SEM. **c** Coronal vibratome sections of P20 brains stained for DAPI. No tumour formation identified in Rescue mice. Tumours (arrowheads) can be found in LATS1/2 cKO, Mutant YAP^f/f^ and Mutant YAP^f/+^ animals. Scale bars = 1 mm.

Although it is generally accepted that YAP1 is the main functional output of the hippo pathway and that its dysregulation leads to tumourigenesis, it remains to be tested whether YAP1 has an essential role in Lats1/2-dependent brain tumour generation. YAP1 exhibits largely redundant functions with its close homologue TAZ. Therefore, we used YAP1 f/f; TAZ f/+ mice^50^ to assess if YAP1/TAZ are required for tumorigenesis in LATS1/2 dual knockout mice. For this purpose, we crossed NEX-Cre; Lats1 f/f; Lats2 f/f mice with YAP1 f/f; TAZ f/+ mice (Fig. 4a). We found that all LATS1/2 cKO animals where one or both copies of YAP1 was deleted still exhibited tumours at P20 (*n* = 3 each), indicating that YAP1 is not the sole downstream factor regulated by LATS1/2. However, we observed that LATS1/2 cKO animals depleted of both copies of YAP1 exhibit a significant reduction (*p* < 0.001, Dunn’s multiple comparisons test) in average tumour size compared with LATS1/2 cKO animals (Fig. 4b), indicating that level of YAP expression impacts tumour size. Further, using a random effects model to account for multiple tumours being recorded from each animal, we observe significant evidence of an additive dosage effect, specifically each YAP1 WT allele lost corresponds to a decrease in estimated tumour volume of ~0.03 mm^3^ (*p* = 0.0068). In LATS1/2; YAP1^f/f^ mice, tumours were negative for YAP1 immunostaining supporting its specificity (Supplementary Fig. 4a). LATS1/2 cKO YAP1^f/+^ TAZ^f/+^ mice have tumours (*n* = 3). However, tumour formation was suppressed at P20 in LATS1/2 cKO YAP1^f/f^ TAZ^f/+^ mice (*n* = 3) (Fig. 4c). These results strongly support that activation of both YAP1 and TAZ mediate tumour formation in LATS 1/2 cKO mice (Fig. 4c). We conclude that YAP1 and TAZ are necessary for LATS1/2-dependent tumourigenesis arising from NEX expression NPCs.

### LATS1/2 cKO tumours recapitulate features of human ependymoma

**Fig. 5:**
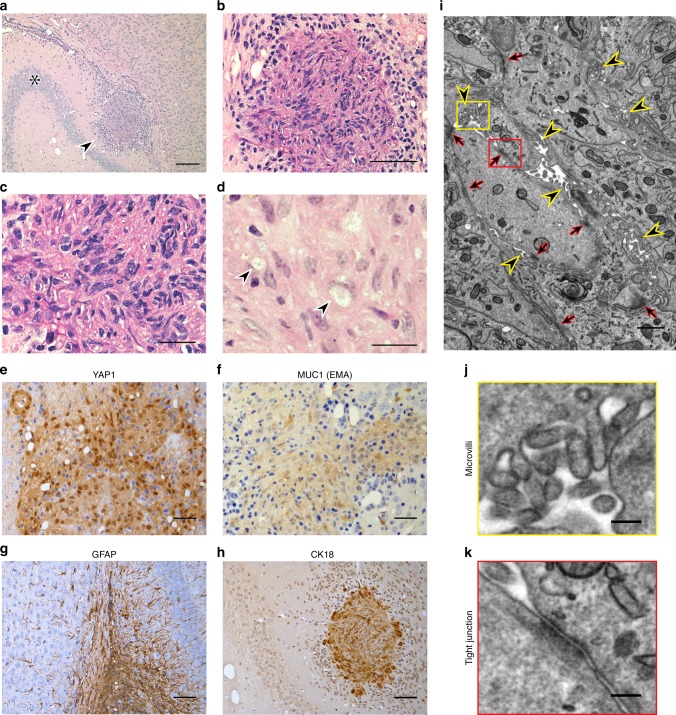
LATS1/2 cKO mouse tumours display histological features of ependymoma. **a**–**d** The tumour (arrowhead) appear to arise from the ependymal layer of the anterior temporal horn of the lateral ventricle at the level of hippocampus (* CA3). **a** H&E, ×4, scale bar = 500 µm; the lesion has compressive margin. **b** H&E, ×10, scale bar = 200 µm and it is composed of fascicles of spindle cells with focal nuclear clustering. **c**. H&E, ×40, scale bar = 50 µm. A few tumour cells contain intracytoplasmic, paranuclear vacuoles (arrowheads). **d**. H&E, ×60, scale bar = 20 µm. Tumour cells demonstrate. **e** nuclear and cytoplasmic YAP1 expression (immunoperoxidase, ×20), **f** weak cytoplasmic MUC1 expression (immunoperoxidase, ×20) and **g** they are focally positive for GFAP (immunoperoxidase, ×20) and **h** cytokeratin 18/CK18 (immunoperoxidase, ×10). Reactive astrocytes surrounding the lesion are also GFAP-positive (**g**—immunoperoxidase, ×20). Scale bars = 50 µm. **i** TEM image (×9900) within the tumour showing multiple ependymal features (Yellow arrowheads = microvilli, red arrows = tight junctions). Scale bar = 1 µm. **j**, **k**. Enlargement of the yellow and red insets in **i**, respectively. Scale bars = 200 nm. **j**. High magnification of microvilli and **k**. a tight junction found in the tumour are shown.

We next investigated the pathological features of the LATS1/2 cKO tumours. Serial sections of paraffin embedded P20 LATS1/2 cKO mice brains showed periventricular multifocal tumours in continuity with the ependymal layer (Fig. 5a). Histologically, the tumours appeared as moderately cellular lesions with a compressive margin. They were composed of uniform spindle or rounded cells with fibrillary or scanty cytoplasm and slightly hyperchromatic nucleus (Fig. 5b, c). We did not observe well-formed perivascular pseudo-rosettes. Some cells contained intracytoplasmic vacuoles (Fig. 5d). Mitoses were absent. The tissue surrounding these lesions showed reactive astrocytosis and microglial response. Tumour cells showed nuclear and lesser extent cytoplasmic YAP1 expression (Fig. 5e), they weakly expressed MUC1 (EMA) (Fig. 5f) and they were focally positive for GFAP (Fig. 5g) and cytokeratin 18 (Fig. 5h). Vimentin, nestin, which are present in radial glia were also expressed in tumours, whereas neuronal marker NeuN was consistently absent (Supplementary Fig. 5a). Ki67 was expressed in tumours indicating cell division (Supplementary Fig. 5a). Notably, normal ependyma in mice also expressed cytokeratin 18 as previously shown^52^ (Supplementary Fig. 5b). Light microscopic features and immunoprofile of the tumours were consistent with ependymoma and similar to the human counterpart^53–55^.

To further test the ependymal origin of LATS1/2 cKO tumours, we performed ultrastructural examination using transmission electron microscopy (TEM). After identifying the areas of the tumour mass using Micro-CT by comparing to nuclear 4′,6-diamidino-2-phenylindole (DAPI) fluorescent image of the brain (Supplementary Fig. 5c), we studied multiple regions of the tumour using serial blockface scanning electron microscopy (Supplementary Fig. 5d) and TEM (Fig. 5i–k, Supplementary Fig. 5e–f). Numerous microvilli and tight junctions (Fig. 5i–k, Supplementary Fig. 5f) were observed in different regions of the tumour. Presence of microvilli and tight junctions confirmed that YAP1 dysregulation and activation leads to the formation of ependymal tumours similar to the corresponding tumours seen in humans.

### RNA sequencing and proteomics in nlsYAP5SA brain

**Fig. 6:**
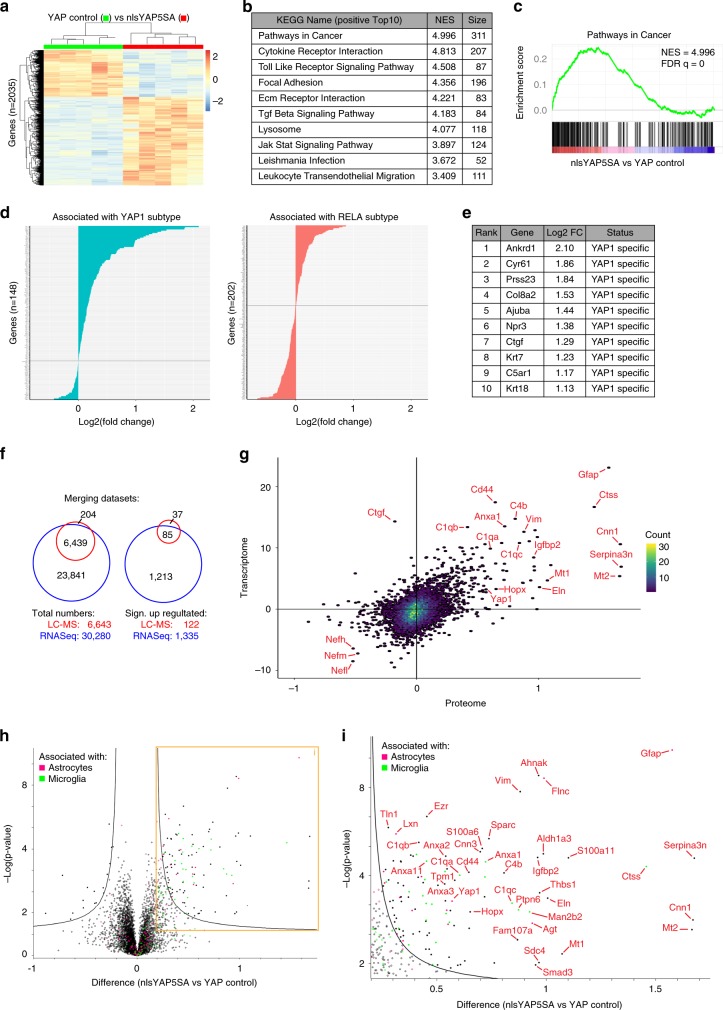
Transcriptome and proteome analysis of nlsYAP5SA mice exhibit further parallels to human ependymoma. **a**–**e** Gene expression analysis for nlsYAP5SA versus control. Five animals per genotype (control = NEX^+/+^ YAP1^nlsYAP5SA/+^; nlsYAP5SA = NEX^Cre/+^ YAP1^nlsYAP5SA/+^) were analysed utilising the Illumina HiSeq 4000. **a** Heat map of all significantly differently expressed genes (2035 genes) between control and nlsYAP5SA animals. Rows represent genes and columns show individual animals. Legend indicates per gene median centred fold change. **b** Gene enrichment analysis (GSEA) of complete gene list (30,280 detected genes) from RNA sequencing (RNASeq) with gene sets derived from the KEGG pathway database. The top 10 annotated pathways with the highest normalised enrichment score (NES) are shown with number of genes in the data set linked to the indicated pathway (Size). **c** GSEA of KEGG annotation “Pathways in Cancer” with highest NES in analysis. **d** Gene list of YAP1 and RELA association (see methods) was used to assign specificity to detected genes in our expression data from nlsYAP5SA screen (350 genes of the 354 in reference list are present in our data set). Log2 fold change of control vs. nlsYAP5SA are ranked from highest to lowest. Teal and red colours are used to denote YAP-specific and RELA-specific genes, respectively. **e** The 10 highest ranked genes with assigned specificity all of these are associated with YAP1. **f** Venn diagrams illustrating numbers of identified genes in Transcriptome (RNAseq, blue) and proteome quantified by liquid chromatography mass spectrometry (LC-MS) (red). Left: all genes, right: significantly upregulated genes in nlsYAP5SA. **g** Density scatter plot of 6439 genes present in both proteome and transcriptome plotted by their expression difference between control and nlsYAP5SA (Welch difference is used for proteomics and stat value is used for transcriptomics). Data sets show a correlation coefficient of 0.5. Each point represents one gene. **h** Volcano plot of difference in protein levels between control and nlsYAP5SA cohort. Each point represents one protein. The *x* axis shows log2 transformed fold change and the *y* axis shows significance by –log10 transformed *p* value obtained by two-sided Welch *t* test. A gene is called significantly and differentially expressed if its FDR is <0.05 and s0 > 0.1, which is indicated by the black line. Proteins associated with astrocytes (magenta) and microglia (green) are displayed. Cell type association was generated by integrating previously published data (see methods). **i** Area shown in **h**, enlarged to display and name significantly upregulated proteins in nlsYAP5SA brains.

We performed total RNA sequencing of the right hemisphere of nlsYAP5SA mice (NEX^Cre/+^; YAP1^nlsYAP5SA/+^) and unaffected littermates (NEX^+/+^; YAP1^nlsYAP5SA/+^) (*n* = 5 for each genotype) (Fig. 6a). Experiments included three additional non-littermate C57BL/6 controls, which clustered with the control animals, but were not included in later analysis (Supplementary Fig. 6a). First, we ranked all 30,280 identified genes based on their differential expression in nlsYAP5SA and control. We then applied gene enrichment analysis (GSEA) choosing the Kyoto Encyclopaedia of Genes and Genomes (KEGG) database to identify gene sets that are overrepresented at both extremes of the ranked list. Among the top 10 upregulated gene sets in nlsYAP5SA we found pathways in cancer, cytokine receptor interaction and focal adhesion, in agreement with active YAP1’s capacity to induce cancer and a consequent immune response (Fig. 6b, c). On the other side, Oxidative Phosphorylation gene set among the most reduced, which may indicate changes in energy metabolism tumours (Supplementary Fig. 6b). In a complementary analysis, we analysed the 2035 significantly differentially expressed genes between the two genotypes utilising the online tool DAVID with KEGG defined gene sets. The significantly differentially expressed gene list was generated using the DESeq2 R package applying the Wald significance test with a threshold of 0.05 for significance. As expected, one of the most-enriched gene sets was the hippo signalling pathway in addition to terms including immune and infection-related genes (Supplementary Fig. 6c). Specifically, in nlsYAP5SA mice numerous known YAP1 downstream effectors^56^ were significantly upregulated at mRNA level, including AMOTL2, CTGF, CYR61, ANKRD1 and AXL (Supplementary Data 1).

Next, we inspected how gene expression signatures in our nlsYAP5SA mouse models compare with published gene expression data from human ependymoma subtypes. For this we used a list of genes that was previously established to be differentially expressed between YAP1-MAMLD1 fusion and RELA fusion-positive human ependymoma subtypes, as well as between YAP1-MAMLD1 and C11orf95-RELA-driven mouse ependymoma models^31,57^. Of these 354 published unique genes (151 YAP1 associated and 203 RELA-associated), 350 were present in our transcriptomics data, which we ranked with respect to fold change in nlsYAP5SA compared with control mice (Fig. 6d). We found that although RELA fusion-associated genes were equally represented among increased and reduced genes in nlsYAP5SA model (Fig. 6d, red), genes that are specific for YAP1-MAMLD1 fusion-positive ependymoma were clearly overrepresented among upregulated genes in the nlsYAP5SA model (Fig. 6d; teal, Supplementary Data 2). When we restricted the analysis to mRNAs that are significantly changed between control and nlsYAP5SA mice (89/354 genes were found in our significantly differentially expressed gene list, matched by gene name) we found that this pattern was even more striking. In all, 45 out of the 46 (98%) YAP1-MAMLD1 fusion-associated mRNAs were significantly increased in our mouse model and only one mRNA was reduced, in contrast 20 out of 43 (46%) RELA fusion-specific genes were increased and 23 were reduced in nlsYAP5SA mice (Supplementary Fig. 6d, Supplementary Data 2). These data show that the proportion of YAP-associated genes that are increased in nlsYAP5SA are much higher than RELA-associated genes (*p* < 0.0001, Chi-squared test), indicating that YAP-MAMLD1 fusion-associated gene expression signatures are present in nlsYAP5SA model. The 18 genes with the highest expression changes in nlsYAP5SA included known YAP1 downstream target genes such as *Ankrd1*, *Cyr61* and *Ctgf* (Fig. 6e)^56^. Interestingly, cytokeratin 18, a marker expressed in the ependymal layer and in our LATS1/2 cKO tumours was among the top 10 increased genes in nlsYAP5SA mice (Fig. 6e). These data support that nlsYAP5SA expression in NPCs lead to a gene expression profile that resembles human ependymomas with YAP1-MAMLD1 fusion. We attempted to perform a principle component analysis between multiple types of paediatric brain tumour gene expression^58^ and our nlsYAP5SA mice-sequencing results. We did not observe a strong clustering of the individual patient tumour types (Supplementary Fig. 6e), thus were not able to compare nlsYAP5SA tumours either, leading this analysis to be inconclusive. Substantial technical caveats were associated with comparing microarray data set with our next-generation sequencing data set (see methods).

To investigate whether the changes we observed in mRNA expression are translated to protein level changes, we took a mass spectrometry approach. The left hemispheres of the nlsYAP5SA and control animals (*n* = 5, each) that were used in the transcriptomic screen were labelled with a TMT (Tandem Mass Tag) 10-plex reagent to enable relative proteome quantification. We identified a total of 6643 proteins, 21.3% of the identified mRNAs were represented at the protein level and we find an overlap of 85 genes and proteins that were significantly increased in both data sets in nlsYAP5SA compared with control when matched by gene name (Fig. 6f). The principal component analysis of the proteomics data shows two distinct clusters clearly separating the different genotypes (Supplementary Fig. 6f). We observed a correlation between the transcript (Stat value) and protein (Welch difference) levels with a Pearson’s correlation coefficient of 0.5 (Fig. 6g, Supplementary Data 3). Next, we calculated categorical enrichment in two dimensions as described previously^59^. Gene ontology (Biological Processes, Molecular Function and Cellular Compartment) and KEGG were annotated both on the transcript and the protein level. These data revealed a good correlation between the transcriptome and proteome data on the categorical level (Supplementary Fig. 6g, Pearson’s *r* = 0.844 correlating 2D enrichment scores). GFAP, vimentin and ANXA1 were specific examples of genes that were increased at mRNA as well as protein levels in nlsYAP5SA brains (Fig. 6g) and these were previously associated with ependymoma^60^.

**Fig. 7:**
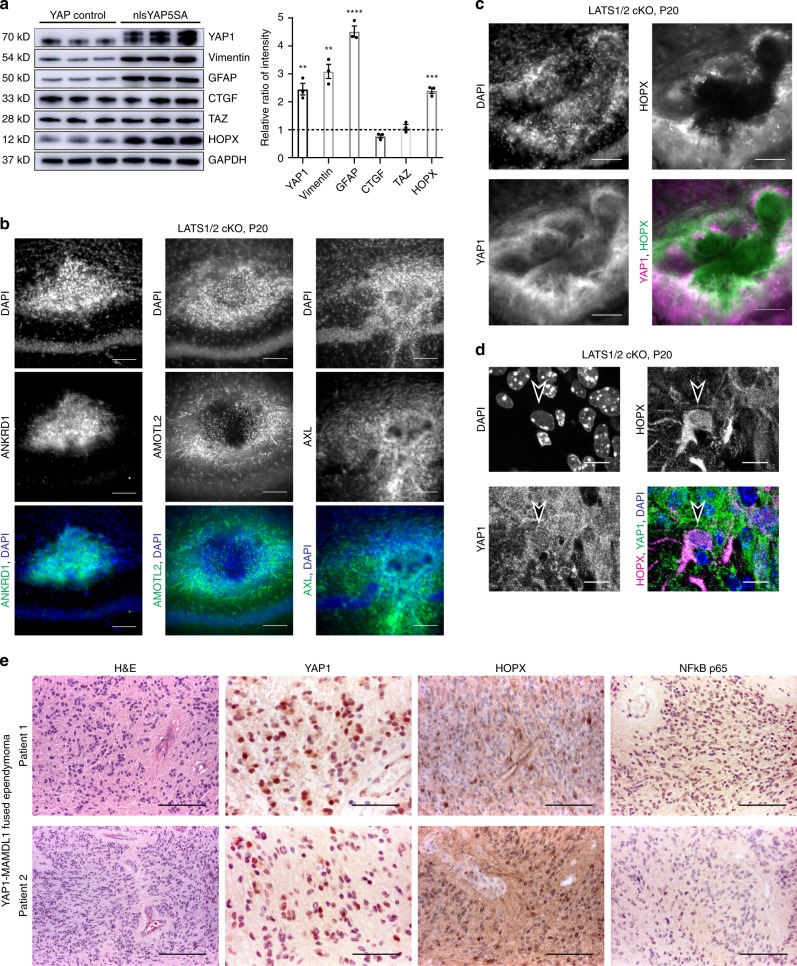
Validation of markers identified in our proteomics and transcriptomics screen in mouse and human models of ependymoma. **a** Western blots and quantification of proteins levels comparing control and nlsYAP5SA animals (control = NEX^+/+^ YAP1^nlsYAP5SA/+^; nlsYAP5SA = NEX^Cre/+^ YAP1^nlsYAP5SA/+^) (*n* = 3 mice, each). Significant increase in YAP1 (*p* = 0.0042), Vimentin (*p* = 0.0017), GFAP (*p* < 0.0001) and HOPX (*p* = 0.0002) are observed in nlsYAP5SA (two-tailed unpaired *t* tests). No significant difference in protein level was observed for TAZ nor CTGF. Error bars = SEM. **b** Immunofluorescence stainings for selected YAP1 downstream genes in LATS1/2 cKO tumours at P20. Strong staining of ANKRD1, AMOTL2 and AXL are observed in the tumour. ANKRD1 was uniformly expressed in throughout all tumour cells, AMOTL2 and AXL exhibited higher levels in tumour near its border. Scale bar = 100 μm. **c** Immunofluorescence of HOPX and YAP1 in a LATS1/2 cKO tumour at P20, showing higher staining at tumour edge, HOPX co-localises with YAP1 in this area (white shows colocalisation, Scale bar = 100 μm). **d** A subset of HOPX expressing cells have clear, nuclear YAP1 staining, example cell shown from a different LATS1/2 cKO tumour (Scale bar = 10 μm). **e** Two YAP1-MAMLD1-fused ependymoma characterised by uniform cells forming pseudo-rosettes in a fibrillary background (H&E, scale bar = 100 μm (Patient 1), 200 μm (Patient 2)); tumour cells show YAP1 nuclear expression (immunoperoxidase, scale bar = 50 μm); there is cytoplasmic and nuclear expression of HOPX (immunoperoxidase, scale bar = 100 μm), whereas p65 is negative in tumour cells (immunoperoxidase, scale bar = 100 μm).

To investigate if increased proteins can be associated with a particular cell type we analysed our proteomics data separately by overlaying it with an existing data set of full proteomics performed on isolated CNS cells^61^. We show that in tumour bearing mice there is an increase in microglia and astrocyte associated proteins (Fig. 6h, i, Supplementary Fig. 6h, i; Supplementary Data 4), indicating a microglial response to the tumour. In addition, complement cascade proteins such as C1qa, C1qb, C1qc and C4b are highly upregulated in agreement with the GSEA pointing towards immune response components (Fig. 6b, Supplementary Fig. 6c). Interestingly, the known YAP1 target CTGF was upregulated in mRNA level but was unchanged at protein level (Fig. 6g), which was further confirmed with western blot (Fig. 7a), suggesting additional posttranscriptional control. Overall, we observed good correlation of our proteomics and transcriptomics analysis, which together strongly support that nlsYAP5S brain tumours resemble YAP1-fusion-positive ependymoma and activate known YAP target genes.

### YAP1 effectors are highly expressed in LATS1/2 cKO tumours

First, we confirmed that Vimentin, GFAP and HOPX were highly increased in nlsYAP5SA brains, where FLAG-tagged YAP1 overexpression can be observed (Fig. 7a). Next, we wanted to know if genes and proteins upregulated in nlsYAP5SA mice were also upregulated in LATS1/2 knockout mouse tumours. Using immunofluorescence, we found that YAP1 downstream effectors identified in mRNA analysis, ANKRD1, AMOTL2 and AXL were increased in LATS1/2 cKO tumours (Fig. 7b). ANKRD1 was abundant in the tumour centre, while AMOTL2 and AXL were higher at periphery, where C3-positive microglia were also observed (Supplementary Fig. 7a). These results suggest that canonical YAP1 signalling effectors are relevant to tumorigenesis of ependymoma-like tumours in both of our mouse models.

In addition to YAP1, Homeodomain-only protein (HOPX) was the only transcription cofactor that was significantly increased in mRNA sequencing and proteomics analysis (Fig. 6g–i). HOPX is a transcriptional regulator expressed in many tissues including neuronal stem cells with astrocytic fate^62,63^. HOPX was present in nucleus as well as cytoplasm, especially at high levels in wild-type mouse cerebellum and dentate gyrus (Supplementary Fig. 7b). HOPX protein was increased in nlsYAP5SA mice (Supplementary Fig. 7c) and in LATS1/2 cKO tumours (Fig. 7c). HOPX co-localised with cells expressing nuclear YAP1 in LATS1/2 cKO tumours (Fig. 7d). These results indicate that HOPX expression is robustly induced in tumour cells with YAP/TAZ activity.

### HOPX is expressed in human YAP1-fusion ependymoma

We next used our proteomics data to predict potential YAP1-fusion ependymoma-specific markers, reasoning that increased protein levels may lead to easier detection with immunostainings. We focused on ANKRD1 and HOPX because upregulated astrocytic and microglial proteins were unlikely to be subtype specific. We used sections of human ependymomas with confirmed YAP1-MAMLD1 and C11orf95-Rela fusions (*n* = 3 patients each). Nuclear YAP1 expression was present in YAP1-MAMLD1 (Fig. 7e), as previously observed^64^, nuclear p65 expression was only observed in C11orf95-RELA-fused ependymoma in keeping with current literature^64,65^ (Supplementary Fig. 7d). Ankrd1 antibody did not yield specific staining IHC. Interestingly, we found that HOPX was expressed in the three YAP1-MAMLD1-induced human ependymoma, while largely absent in three C11orf95-RELA-induced ependymoma (Fig. 7e, Supplementary Fig. 7d). HOPX has been reported to be absent or suppressed in malignancies^63,66^ and has been identified as a significant enhancer in YAP1-fused ependymomas^67^. It is possible to speculate that HOPX expression may impact on the more favourable outcomes of YAP1-fusion ependymoma^32^. These first results will be followed up by larger cohorts in future studies.

## Discussion

Our study demonstrates that control of YAP1/TAZ activity in neuronal precursor cells at VZ by LATS1/2 kinases is critical for limiting their proliferation. Upon nlsYAP5SA expression, hippocampal neuron differentiation is severely affected. These results are in line with the findings that NF2, a positive regulator of hippo signalling, also alters hippocampal formation by limiting progenitor numbers and enabling hippocampal neuron differentiation; effects mediated by YAP1^68,69^. Our LATS1/2 cKO mouse model data indicate that LATS kinases play a role in suppressing YAP1 activity in progenitors, thus establishing a requirement for the canonical LATS-YAP1 signalling pathway in limiting progenitor proliferation.

Mouse models of ependymoma are important for understanding the pathogenesis of this tumour and its subtypes and developing therapeutic interventions. Alongside patient-derived xenografts^70,71^, mouse models of ependymoma that mimic genetic causes in cells of origin are invaluable for studying tumour development^57,72,73^. For example, isolated cerebral embryonic neural stem cells from Ink4a/Arf-deleted mice, a genetic aberration previously observed in human ependymoma^33^ were transformed by an Ephb2-expressing virus^72^. This mouse model, mEP^Ephb2re^, was used in high-throughput screens, which identified 5-fluoroucil as an effective treatment^74^, a hypothesis that is being tested clinically^75^. More recently, a mouse model, RELA^*FUS1*^ was generated by transducing nestin expressing mouse stem cells with a c11ORF95-RELA fusion protein^73^. In both mouse models, upon engrafting, tumours with profound similarities to ependymoma were formed^72,73^. Similar to other ependymoma mouse models^57,72^, in LATS1/2 cKOs we did not observe rosettes or well-formed pseudo-rosettes, which are commonly observed in human ependymomas and are also present in the mEP^Ephb2^ model^71^. We speculate that formation of these structures may depend on tumour size and species.

The tumours generated by increased YAP/TAZ activity in our nlsYAP5SA and LATS1/2 cKO mouse models are located in the periventricular region, similarly to ependymoma with YAP1-MAMLD1 fusion in humans, which consistently show a periventricular location, with or without an intraventricular component^32^, in contrast with RELA-fused ependymoma, which tend to occur more often in cortical-centred location^64^. A recent publication showed that YAP1-MAMLD1 fusion in nestin-positive neural stem cells induce ependymoma-like tumours in mice^57^. In this model, MAMLD1 was necessary for the nuclear localisation of YAP1 and confers specific properties to YAP1-dependent gene regulation^57^. Our nlsYAP5SA and LATS1/2 cKO mouse models indicate the notion that YAP/TAZ activity alone is sufficient for ependymoma-like tumour formation. This result supports that the main function of fusion proteins such as MAMLD1 may be nuclear localisation and/or activation of YAP/TAZ. Importantly, by using transcriptomics analysis, we found that nlsYAP5SA brains have similar gene expression patterns to YAP1-MAMLD1 fused ependymoma and YAP1-MAMLD1 fusion induced mouse model^57^. We were not able to demonstrate a clustering of our nlsYAP5SA gene expression data with ependymoma, when using a data set with multiple paediatric brain tumour microarray analysis^58^. To establish the relationship of nlsYAP5SA mouse model to paediatric tumours in general and strengthen it as a preclinical model, it would be necessary to compare our mRNA sequencing data set with next-generation sequencing data sets of paediatric brain tumours, including YAP1-fusion ependymoma. Using genetic rescues by deleting YAP1 and TAZ from the same cells with LATS1/2 deletion, we demonstrated that either of these paralogs can lead to tumour formation. Our results show that increased YAP/TAZ activity is sufficient to ependymoma-like tumours in mice.

It was advantageous in this study to combine transcriptomics with proteomics, as some mRNA changes may not be reflected in protein levels. Our proteomics screen was able to detect ~21% of genes detected in our RNA sequencing screen, with the highest proteomic hits indicating high protein abundance and therefore potential use as histological markers for diagnosis and therein prognosis. With this reasoning we tested HOPX in IHC and showed high level of expression in tumours with YAP1-MAMLD1 fusion. Future studies in large cohorts of ependymoma subtypes would be needed to assess subtype-specificity of this marker. Nevertheless, these data support the use of nlsYAP5SA and LATS1/2 cKO mice as ependymoma models.

Our proteomics screen showed a striking increase in microglia-associated proteins, such as the ANXA1 and complement factors. Genomics signatures from whole tissues would inevitably contain signatures of all cellular types. High-throughput tissue staining would be needed to clarify cellular subtypes in tumours. In our LATS1/2 cKO mouse model, although some of the tumour markers were highly present in the centre of the tumour mass, e.g., ANKRD1, Vimentin, CK18, nestin and Ki67, some were localised towards the edges of the tumour, e.g., HOPX, AMOTL2, AXL and microglial marker C3. For the latter category, further studies would be needed to distinguish between a cell autonomous effects of YAP1 expression in Cre recombined cells from an effect of YAP1-induced tumours on the surrounding wild-type cells.

Our results show that nlsYAP5SA and LATS1/2 cKO mouse models are similar to ependymoma in general, in terms of histology, immunoprofile and ultrastructural features. Specifically, nlsYAP5SA mouse tumours highly resemble YAP1-fusion ependymoma in gene expression. Our study suggests that nlsYAP5SA and LATS1/2 cKO mice are valuable models of YAP1-fusion ependymoma for biological and preclinical research. Small tumour sizes, early lethality and lack of pseudo-rosettes may have limitations. Complementary models, such as xenografts, could be used in conjunction with genetically defined mouse ependymoma models, to reach a more comprehensive understanding of ependymoma.

## Methods

### Human tissue and staining

Tissue of six patients were used in this study. Written consent was given by the patient (one adult RELA tumour) or by legal representatives (five children, three YAP and two RELA ependymoma). All tumours at diagnosis without any previous treatments such as chemotherapy nor radiotherapy.

For IHC tissue analysis of YAP1, HOPX and RELA expression, the patient tissues were deparaffinised and subject to Heat-Mediated Antigen Retrieval (pH6 Citric Buffer, microwave 23 min). The slides were incubated with YAP1 (1:400, Cell Signalling #14074), HOPX (1:250, Proteintech #11419-1-AP), NF-kB p65 (1:800, Cell Signalling #8242) in signal stain diluent overnight at 4 °C and 45 min in secondary antibody. Diaminobenzidine was utilised as the chromogen and the sections were counterstained with haematoxylin. Images were acquired with a Leica DM2500 LED.

### Animals

Maintenance and handling of animals were performed under regulations of the Animal (Scientific procedures) Act 1986 of the United Kingdom and approved by institutional ethical reviews. Mice were group housed and maintained on a 12 hr light/dark cycle, with food and water provided ad libitum. None of the experimental mice were immune compromised. Both male and female mice were used and randomly allocated to experimental groups according to genotypes. LATS1/2 cKO and nlsYAP5SA mice were closely monitored and weighed regularly. The majority of LATS1/2 cKO mice were collected at <3 weeks of age. In accordance to our project license, a subset of LATS1/2 cKO animals were aged up to 2 months of age, provided they did not exceed 10% weight loss criteria (~50% were collected before reaching 1 month of age to prevent reaching this end point) and nlsYAP5SA mice were aged only up to 2 weeks owing to higher severity.

Nex-Cre (Neurod6^tm1(cre)Kan^, MGI:2668659) was a generous gift from Dr. Klaus Nave. *Lats1*^*f/f*^ *Lats2*^*f/f*^ Lats1(tm1.1Jfm) MGI:5568586 and Lats2(tm1.1Jfm) MGI:5568589 mouse was kindly provided by Dr. R. Johnston (University of Texas MD Anderson Cancer Center, USA). Ai14 (Gt(ROSA)26Sor^tm14(CAG-tdtomato)Hze^) was purchased from Jackson Laboratories (Stock Number:007908). YAP1^f/f^ (Yap1^tm1c(KOMP)Mbp^, MGI:5603606), TAZ^f/f^ (Wwtr1^tm1c(EUCOMM)Wtsi^, MGI:5603602) mice were from Barry Thompson’s laboratory and nlsYAP5SA mice were described previously^45^.

### Tissue preparation, immunofluorescence and imaging

For brain tissue section staining, P0 mice were decapitated and the brain fixed in 4% ice-cold paraformaldehyde (PFA) in PBS for 2 days at 4 °C. Older animals were transcardial perfused with 4% ice-cold PFA under terminal anaesthesia. Brains were dissected out and post-fixed in 4% PFA overnight at 4 °C. For immunofluorescence, coronal or sagittal sections were prepared with a vibratome at 50 µm. The sections were blocked with 10% serum and 0.2% Triton-X in PBS and incubated with primary antibody in blocking solution overnight at 4 °C. Used antibodies include AMOTL2 (1:100, GeneTex # CTX120712), ANKRD1 (1:100, Proteintech #11427-1-AP), AXL (1:100, R&D Systems #AF854), C3 (1:100, Abcam #ab11862), Cre (1:500, Covance #PRB-106P), Ctip2 (1:500, Abcam #ab18465), GFAP (mouse 1:500, Sigma #G6171 and chicken 1:1000, Abcam #ab134436), HOPX (1:100, Proteintech #11419-1-AP), Ki67 (1:100/1:300, BD #550609), MUC1 (1:500, Abcam #ab45167), Nestin (1:100, Millipore #MAB353), NeuN (1:100, Millipore #MAB377) and YAP1 (rabbit 1:100, CST #14074 and mouse 1:100, Santa Cruz Biotechnology #sc-101199). Sections were incubated with the appropriate Alexa fluor 488-, 546- or 647-conjugated secondary antibodies (1:500; ThermoFisher) for 2 hr at room temperature. Nuclei were stained using DAPI. Sections were mounted on slides with Fluoromount. Images were acquired with Olympus IX83 or a Leica MPSP5 Upright Confocal as indicated in the figure legends.

### Histology and IHC

Brains subject to histological analysis were dehydrated and embedded in paraffin after completion of 4% PFA fixation. The tissues were sectioned (4 µm) and sections were prepared for H&E or IHC. Unless stated otherwise the heat induced antigen retrieval (citrate buffer pH = 6, 23 min microwave) has been applied and primaries were incubated for 1 hr at RT. The following antibodies were used for IHC; CK18 (1:3000, ThermoFisher #PA5-14263; Tris-EDTA pH9), GFAP (1:750, DAKO #z0334), HOPX (1:250, Proteintech #11419-1-AP), Ki67 (1:350, Abcam #ab16667), MUC1 (1:500, Abcam #ab15481), Nestin (1:600, BD Biosciences #611659), Rela/NF-kB p65 (1:800, Cell Signalling #8242), (NeuN (1:600, Chemicon #MAB377), Vimentin (1:600, Abcam #ab92547), YAP1 (1:400, CST #14074; in signal stain (from CST) overnight at 4 °C). Slices were imaged using the Olympus VS120 Slide Scanner and/or the Leica DM2500 LED.

### Electron microscopy

For serial blockface scanning electron microscopy (SBF SEM) and TEM, mouse brains were perfused fixed in 2% PFA, 2.5% GA in 0.1 M PB pH 7.4 and post-fixed for at least 4–5 hours in 2% PFA in 0.1 M PB pH 7.4 at 4 °C. The brains were then sectioned using a Leica VT1000 S vibrating blade microtome (Leica). In all, 50 and 100 µm sections were collected and stored in 0.1 M PB. The 50 µm sections were then stained with DAPI and imaged as previously to identify the locations of the tumours. The consecutive 100 µm sections were then transferred to polypropylene 24-well plates (Caplugs Evergreen, Buffalo, USA) and processed using a Pelco BioWave Pro+ microwave (Ted Pella Inc, Redding, USA) and following a protocol adapted from the National Centre for Microscopy and Imaging Research protocol (Latest version available: https://ncmir.ucsd.edu/sbem-protocol). Each step was performed in the Biowave, except for the PB and water wash steps, which consisted of two washes on the bench followed by two washes in the Biowave without vacuum at 250 W for 40 s. All the chemical incubations were performed in the Biowave for 14 min under vacuum in 2 min steps alternating with/without 100 W power. The SteadyTemp plate was set to 21 °C unless otherwise stated. In brief, the samples were fixed again in 2.5% glutaraldehyde (TAAB)/4% formaldehyde in 0.1 M PB. The cells were then stained with 2% osmium tetroxide (TAAB)/1.5% potassium ferricyanide (Sigma), incubated in 1% thiocarbohydrazide (Sigma) with SteadyTemp plate set to 40 °C, and further stained with 2% osmium tetroxide in ddH_2_O. The cells were then incubated in 1% aqueous uranyl acetate (Agar Scientific, Stansted, UK) with SteadyTemp plate set to 40 °C, and then washed in dH2O with SteadyTemp set to 40 °C. Samples were then stained with Walton’s lead aspartate with SteadyTemp set to 50 °C, and dehydrated in a graded ethanol series (20%, 50%, 70%, 90% and 100%, twice each), followed by three dry acetone washes at 250 W for 40 s without vacuum. Exchange into Durcupan ACM resin (Sigma) was performed in 25%, 50% and 75% resin in acetone, followed by 4 pure Durcupan steps, at 250 W for 3 min, with vacuum cycling (on/off at 30 sec intervals), before embedding at 60 °C for 48 h. Blocks were then mounted for micro-computed tomography (micro-CT)) on a cylindrical specimen holder with Devcon S-210 epoxy glue. The mounting was done with the longitudinal axis of the brain section oriented vertically. Tomographic imaging was conducted in an Xradia Versa 510 (Carl Zeiss Ltd, Cambridge, UK). A low resolution scan was captured at xkV/xW, with *x* projections and a pixel size of *x*. The region of interest was identified using 3DXMViewer software (Zeiss) and co-ordinates located for positioning in the Xradia Versa 510. A high-resolution scan was captured at xkV/xW, with *x* projections and a pixel size of *x*. The data were exported as tiff, to be visualised in 3D in ClearVolume, a plug-in of the FIJI framework^76^. The samples were then trimmed to a small trapezoid, excised from the resin block, and attached to a SBF SEM specimen holder using conductive epoxy resin (Circuitworks CW2400). Prior to commencement of a SBF SEM imaging run, the samples were coated with a 2 nm layer of platinum to further enhance conductivity.

SBF SEM data were collected using a 3View2XP (Gatan Inc., Pleasanton, CA) attached to a Sigma VP SEM (Carl Zeiss Ltd). Inverted backscattered electron images were acquired through the entire extent of the region of interest. For each 50 nm slice, a low resolution overview image (horizontal frame width 800 µm for 02/384 µm for 03; pixel size of 78 nm for 02/48 nm for 03; using a 2 µs dwell time) and several high-resolution images of the different regions of interest (horizontal frame width 170 µm for 02/183 µm for 03; pixel size of 8.3 nm for 02/11.4 nm for 03, using a 2 µs dwell time) were acquired. The SEM was operated in variable pressure mode at 5 Pa. The 30 µm aperture was used, at an accelerating voltage of 2.5 kV for 03, 2 kV for 02. In total, 130 (for 02) and 400 (for 03) slices were necessary to get enough 3D information for an entire tumour. As data were collected in variable pressure mode, only minor adjustments in image alignment were needed. All the images were converted as tiff in Digital Micrograph (Gatan Inc.), and the tiff stacks were automatically aligned using TrakEM2, a plug-in of the FIJI framework^77^.

After SBF SEM, the samples were serial sectioned using a UC7 ultramicrotome (Leica Microsystems, Vienna, Austria) and 70 nm sections were picked up on Formvar-coated 2 mm slot copper grids (Gilder Grids Ltd., Grantham, UK). The first sections were viewed using a 120 kV Tecnai G2 Spirit transmission electron microscope (FEI Company, Eindhoven, Netherlands) and images were captured using an Orius CCD camera (Gatan Inc.).

### Tissue collection from mice

For RNA sequencing and mass spectrometry control and nlsYAP5SA littermates from two matings were collected at the age of P11 (five animals per genotype). In additio, three age-matched C57BL/6 animals where collected. The pups were killed by cervical dislocation, decapitated and the brain dissected out of the skull. Olfactory bulbs and cerebellum were split off and the hemispheres were separated. Although the right hemisphere was put into RNAlater (Qiagen, #76104), the left hemisphere was snap frozen in liquid nitrogen.

For western blotting, control and nlsYAP5SA were collected at the age of P11 (three animals per genotype). The pups were killed by cervical dislocation, decapitated and the brain dissected out of the skull. As before the olfactory bulbs and cerebellum were split off and the hemispheres were separated, flash freezing the right hemisphere in liquid nitrogen.

### RNA extraction and sequencing

RNA of tissue stabilised in RNAlater was extracted using the RNeasy Mini Kit (Qiagen #74104) according to manufacturerʼs protocol. The samples were quantified via GloMax (Promega) and RNA quality evaluated using an RNA ScreenTape on the Agilent TapeStation. All RNA samples had RIN scores above 7. The libraries were prepared with the KAPA mRNA HyperPrep kit (Roche), normalising samples to 500 ng RNA input and prepared these into Illumina compatible libraries according to the kit manufacturer’s instructions. The libraries were normalised and pooled for sequencing. Sequencing was carried out on the Illumina HiSeq 4000, where the pool was mirrored across 2.5 lanes—yielding an average of 27 million reads per sample (±million reads). Sample QC, library preparation and sequencing were carried out with the advanced sequencing facility at the Francis Crick Institute.

### RNA sequencing data analysis

Raw fastq files were adapter trimmed using CutAdapt 1.5^78^ and these trimmed reads were mapped to GRCm38 release 86 with associated ensemble transcript definitions using STAR 2.5.2a^78^ wrapped by the R package RSEM 1.3.0^79^, which was used to calculate estimated read counts per gene. Where necessary, bam files were merged using samtools 1.8.^80^. Estimated counts were normalised and differentially expressed genes were called between genotype group using the R package DESeq2 1.12.3.^81^. Genes with an adjusted *p* value ≤ 0.05 were said to be differentially expressed. Heatmaps were generated using the R package pheatmap 1.0.8 https://CRAN.R-project.org/package=pheatmapall analyses were performed using R 3.3.1 (R Core Team “R: A Language and Environment for Statistical Computing” https://www.R-project.org/). GSEA of complete pre ranked gene list was done using GSEA 4.0.1^82^ set to classic enrichment statistics utilising the c2.cp.kegg.v7.0.symbols.gmt gene matrix containing 186 gene sets. Number of permutations set to 1000, classic enrichment statistic with 15 and 500 set as minimum and maximum, respectively, for set inclusion.

Significantly differentially expressed gene list was uploaded to DAVID 6.8^83,84^ with 44.8% (887) found annotated in KEGG Pathway.

To investigate the resemblance of nlsYAP5SA mouse model to human ependymoma subtypes, we utilised previously reported microarray data on differentially expressed genes between human ST-EPN-YAP1 and ST-EPN-RELA subtypes, which were also present and differentially expressed in YAP1-MAMLD1 induced and C11orf95-RELA induced mouse models of ependymoma^31,57^. The summarised synthesis of these subtype-associated, differentially expressed genes listed in Supplementary Data 6 from ref. ^57^ was compared with our complete gene list as well as our significantly differentially expressed gene list. Log2 fold change of our data set was plotted and the identified of specification was taken from the other data set using R 3.5.1.

For comparison of the nlsYAP5SA gene expression profile with a previously published human peadiatric brain tumours data set containing; ependymoma, glioblastoma, medulloblastoma and pilocytic astrocytoma^58^. Adjustments made prior to the comparative analysis: the human Affymetrix microarray report 21,050 probes of which 10,347 (49.2%) have a corresponding Ensemble identifier using the latest version of the Bioconductor annotation (hgu113aENSEMBL2PROBE). Of these, 5771 (27.4%) are assigned a gene symbol with an annotated mouse homologue with a well-defined Ensemble identifier. Almost all of these 5750 are included in the annotation that we use for the RNA-sequencing analysis. In all, 68 of these had duplicates leaving a total of 5682 RNA-sequencing probes from the original 21,050 gene probes of the Affymetrix microarray. In total, 5682 gene expression data (27% of initial Affymetrix probes) were compared with our sequencing results from Illumina. Normalisation was performed gene-wise: each data set was individually normalised by median centering and scaling by the standard deviation for each gene. These scaled data sets were then merged and the same median centering and standard deviation scaling applied, per gene, to this merged data set. The heat map was generated with the R package pheatmap in R 3.3.1 (details see above). Substantial reduction of the data set and normalisation protocols described above constitute considerable caveats for this experiment.

### Mass spectrometry and data processing

Liquid chromatography‐tandem mass spectrometry (LC‐MS/MS) was used to analyse differences in protein expression in P11 nlsYAP5SA compared with control animals (*n* = 5 each). The left hemisphere of each animal was taken and lysed in lysis buffer containing 8 M Urea, 50 mM HEPES pH8.2, 10 mM Glycerol 2-phosphate, 50 mM sodium fluoride, 5 mM sodium pyrophosphate, 1 mM EDTA, 1 mM sodium vanadate, 1 mM dithiothreitol (DTT), 1× Protease Inhibitor Cocktail (Roche), 1× Phosphateae inhibitor Cocktail, 1 µM okadaic acid and incubated for 30 minutes on ice. Protein concentration was determined with BCA protein assay according to manufacture instructions. 100 µg per samples was reduced with DTT, alkylated with IAA, quenched with DTT and digested with rLysC (Lysyl Endopeptidase, Mass Spectrometry Grade (Lys-C), 21-05063, Lot#CAR2347) and Trypsin (Pierce Trypsin Protease, MS Grade, 90058, Lot#TK276718). After acidification with trifluoroacetic acid (TFA) the samples were desalted on C18 MacroSpin columns (Nest Group) followed by freeze drying them in liquid nitrogen prior to drying them on the speed vac. Subsequently the samples were labelled with a TMT 10-plex hit (ThermoFisher, 90110, Lot#TJ268160) according to manufacturer protocol. TMT labelling efficiency was confirmed (>99%) and mixing check was performed prior to quenching the labelling reaction. The samples were combined, partially dried and then cleaned on a C18 SepPak and aliquoted. Subsequently one-tenth of the sample was resolubilised and fractionated into eight fractions utilising the Pierce High pH Reversed-Phase Peptide Fractionation Kit (ThermoFisher #84868). All samples were dried and resolubilised in 0.1% TFA prior to LC-MS/MS analysis. Peptides were separated on a 50 cm, 75 µm I.D. Pepmap column over a 180 min gradient and eluted directly into the mass spectrometer (Orbitrap Fusion Lumos ETD) with HCD MS2 fragmentation and in a second injection (7 µl each) analysed in MS3. Xcalibur software was used to control the data acquisition. The instrument was run in data-dependent acquisition mode with the most abundant peptides selected for MS/MS by HCD fragmentation. MaxQuant v1.6.6 was used to process the raw data acquired with a reporter ion quantification method. Adjusted reporter ion isotopic distributions according to the TMT Lot number. Protein database searching was done by Andromeda search engine using the Uniprot KB database of mus musculus sequences (dated 08 Jan 2016). The protein group table was uploaded into Perseus 1.4.0.2 for subsequent statistical analysis and data visualisation. A two-sided Welch *t* test with threshold set to FDR = 0.05 and s0 = 0.1 was used to display significance.

To investigate association of proteins to particular cell types the data were merged with existing data sets from^61^. From this publication, Supplementary Data 15 were used to identify protein association to isolated cell types and Supplementary Data 8 were used to include data from cultured cell types. The data sets were partially merged (by matching gene name, integrating the log2 fold expression columns per cell type of the other data set into ours) and association to particular isolated CNS cell type was defined by expression value being a minimum of three in one of the four isolated cell types (astrocytes, microglia, neurons and oligodendrocytes) and with a value of at least double in one compared with any of the other cell types.

### Merging transcriptome and proteome data

The total RNA and protein lists were merged by gene name and plotted using the stat and Welch difference values respectively. Annotation was done in Perseus 1.4.0.2 using annotation sets KEGG, Gene Ontology Cellular Compartment, Molecular Function and Biological Process. 2D annotation of the proteome and transcriptome was done using Benjamini-Hochberg FDR as truncation and a threshold value of 0.02.

### Western blots

Flash frozen Mouse brains were homogenised by sonication in sample buffer containing 0.2 M DTT. Lysates were centrifuged at 13,000 *g* for 15 minutes and supernatants denatured at 95 C for 10 minutes. Equal amount of protein from the brain lysates were ran on a NuPAGE 4–12% Bis-Tris polyacrylamide gels (Invitrogen) and then transferred to a polyvinylidene difluoride membrane (Millipore). After blocking in 5% non-fat milk in TBST for 60 minutes, the membrane was incubated with the primary antibodies overnight at 4 °C. The following antibodies were used; CTGF (1:1000, Abcam, #ab6992); CYR61 (1:1000, Abcam, #ab24448); GAPDH (1:5000, Abcam, #ab8245); GFAP (1:1000, Sigma-Aldrich, #G6171); HOPX (1:1000, ProteinTech, #11419-1-AP); TAZ (1:1000, Abcam, #ab84927); Vimentin (1:5000, Abcam, #ab92547) and YAP1 (1:2000, Cell Signalling, #14074). Subsequently, membrane were incubated with the horseradish peroxidase-conjugated (HRP) secondary antibody for 60 minutes—HRP-conjugated mouse (1:10,000; Jackson #715-035-151) and HRP-conjugated rabbit (1: 10,000; Jackson #711-035-152). Finally, the membrane was developed with ECL system (Pierce) and visualised by a chemiluminescence detection system (Amersham WB System). Western blot analysis was performed using ImageJ software (version 1.52r).

### Tumour measurements

Three animals of LATS1/2 cKO, Mutant^f/+^, Mutant^f/f^ and rescue were subject to detailed tumour size analysis. The brains were sectioned coronal using the vibratome with 50 µm depth. Every second section was stained with a tumour marker (Vimentin, YAP1 or GFAP) to aid tumour identification and DAPI. All sections were evaluated and imaged using the Olympus IX83. The area of each identified tumours was measured in all sections using FIJI and volume was calculated by section thickness and taking gap sections into account. Kruskal–Wallis test was performed to evaluate if the tumour size is significantly different from any group to another (*p* = 0.0009). Post hoc the Dunn’s multiple comparisons test was performed to identify which groups are significant from each other.

In addition, the effect of change of genotype on estimates volume was determined by encoding genotype as an ordered factor (Mutant^f/f^ < Mutant^f/+^ < LATS1/2 cKO) and fitting a linear mixed effects model with animal as the random effect using the function glmmPQL from the MASS package with the call “glmmPQL(vol ~ geno, random = ~1|animal, “gaussian”)” diagnostic plots from lm(vol ~ geno/animal) indicate that normality of residuals and heteroscedasticity were reasonable assumptions but the mixed effects function was used for hypothesis testing to account for “proper” nesting of animals within genotypes. The quadratic term for genotype was non-significant (*p* = 0.8764).

### Statistical analysis and reproducibility

Statistical analysis was performed using GraphPad Prism 7 software, R 3.3.1 or R 3.5.1 using statistical tests as indicated in the text. Statistical significance was visualised as: ns = not significant (*p* > 0.05), * = *p* < 0.05, ** = *p* < 0.01, *** = *p* < 0.001, **** = *p* < 0.0001.

All immunostaining experiments have been done using three mice and representative images have been chosen for figures. For TEM, which has been conducted on two animals due to the resource limitations; all features (microvilli, tight junctions) observed in LATS1/2 cKO mice have been found throughout the tumours in both mice.

### Reporting Summary

Further information on research design is available in the Nature Research Reporting Summary linked to this article.

## Supplementary information

Supplementary Information

Description of Additional Supplementary Files

Supplementary Data 1

Supplementary Data 2

Supplementary Data 3

Supplementary Data 4

Reporting Summary

## Data Availability

mRNA sequencing of nlsYAP5SA brains generated in this study have been deposited in National Center for Biotechnology Information (NCBI) Gene Expression Omnibus (GEO) under GSE147961 [https://www.ncbi.nlm.nih.gov/geo/query/acc.cgi?acc=GSE147961]. The quantitative proteomics (TMT) data set of nlsYAP5SA brains is deposited in Pride under PXD018131. The YAP1-MAMDL1 and C11orf95-RELA gene expression data to determine YAP1/RELA specificity referenced in our study is from Pajtler et al. Supplementary Data 6^57^. The paediatric brain tumour gene expression data set used in Supplementary Fig. 6e is from^58^ deposited at NCBI GEO database and are publicly accessible under accession number GSE50161. The data set used for cell type association was taken from Sharma et al. Supplementary Data 8 and 15^61^.

## Source data

Source Data
