# Author Correction: YAP1/TAZ drives ependymoma-like tumour formation in mice

**DOI:** 10.1038/s41467-020-18851-5

**Published:** 2020-09-28

**Authors:** Noreen Eder, Federico Roncaroli, Marie-Charlotte Domart, Stuart Horswell, Felipe Andreiuolo, Helen R. Flynn, Andre T. Lopes, Suzanne Claxton, John-Paul Kilday, Lucy Collinson, Jun-Hao Mao, Torsten Pietsch, Barry Thompson, Ambrosius P. Snijders, Sila K. Ultanir

**Affiliations:** 1 Kinases and Brain Development Laboratory, The Francis Crick Institute, London, NW1 1AT UK; 2 Protein Analysis and Proteomics Platform, The Francis Crick Institute, London, NW1 1AT UK; 3 Manchester Centre for Clinical Neuroscience, Salford Royal NHS Foundation Trust, Salford and Division of Neuroscience and Experimental Psychology, Faculty of Biology, Medicine and Health, School of Biology, University of Manchester, Manchester, M13 9PT UK; 4 Electron Microscopy Platform, The Francis Crick Institute, London, NW1 1AT UK; 5 Bioinformatics and Biostatistics Platform, The Francis Crick Institute, London, NW1 1AT UK; 6 Institute of Neuropathology, DGNN Brain Tumour Reference Center, University of Bonn, Bonn, Germany; 7 Centre for Paediatric, Teenage and Young Adult Cancer, Faculty of Biology, Medicine and Health, University of Manchester, Manchester, M13 9PT UK; 8 Department of Molecular, Cell and Cancer Biology, University of Massachusetts Medical School, Worcester, MA 01605 USA; 9 Epithelial Biology Laboratory, The Francis Crick Institute, London, NW1 1AT UK

**Keywords:** Cancer models, CNS cancer, Tumour-suppressor proteins, Phosphorylation, Cell proliferation

Correction to: *Nature Communications* 10.1038/s41467-020-16167-y, published online 13 May 2020.

The original version of this article contained an error in the spelling of the author Marie-Charlotte Domart, which was incorrectly given as Marie-Charlotte Dolmart.

This has now been corrected in both the PDF and HTML versions of the article.

