# Supplementary material for: YAP1/TAZ drives ependymoma-like tumour formation in mice
Source: Nat Commun. 2020 May 13;11:2380. doi: 10.1038/s41467-020-16167-y (PMC7220953; doi:10.1038/s41467-020-16167-y)
Supplement: Supplementary file 2 — Description of Additional Supplementary Files [file 41467_2020_16167_MOESM2_ESM.pdf]

Description of Additional Supplementary Files

File Name: Supplementary Data 1
Description: All 2035 significantly differentially expressed genes found in nlsYAP5SA mice brain when compared to YAP control animals. Ensemble identifier, gene name, stat value and adjusted p-value are included.

File Name: Supplementary Data 2
Description: Merged list of genes of our transcriptome dataset which were found mentioned in the Pajtler *et al* dataset with a YAP1- or RELA- specificity. Ensemble identifier, gene name, log2 fold change, YAP1-/RELA- specificity and if the RNA is part of the significantly differentially expressed gene list (nlsYAP5SA vs YAP control brains) is indicated.

File Name: Supplementary Data 3
Description: Merged dataset of genes identified in both the transcriptome and the proteome screen. Gene name, stat value (transcriptome), welch difference (proteome) and UniProt identifier are included.

File Name: Supplementary Data 4
Description: Our proteome dataset merged by gene name with Sharma *et al* isolated central nervous system cell types protein list. Association to particular isolated CNS cell type was defined by expression value being a minimum of 3 in one of the four isolated cell types (astrocytes, microglia, neurons and oligodendrocytes) and with a value of at least double in one compared to any of the other cell types. Indication if protein is part significantly differentially expressed in nlsYAP5SA when compared to YAP control, Gene name, indication if protein is associated to one of the cell types, protein ID, majority protein ID and protein name are included.
