# Supplementary material for: YAP1/TAZ drives ependymoma-like tumour formation in mice
Source: Nat Commun. 2020 May 13;11:2380. doi: 10.1038/s41467-020-16167-y (PMC7220953; doi:10.1038/s41467-020-16167-y)
Supplement: Supplementary file 7 — Reporting Summary [file 41467_2020_16167_MOESM7_ESM.pdf]

Corresponding author(s): Sila Ultanir sila.ultani@crick.ac.uk

Last updated by author(s): Feb 4, 2020

# Reporting Summary

Nature Research wishes to improve the reproducibility of the work that we publish. This form provides structure for consistency and transparency in reporting. For further information on Nature Research policies, see Authors & Referees and the Editorial Policy Checklist.

## Statistics

For all statistical analyses, confirm that the following items are present in the figure legend, table legend, main text, or Methods section.

| n/a | Confirmed | |
|---|---|---|
| ☐ | ☒ | The exact sample size (*n*) for each experimental group/condition, given as a discrete number and unit of measurement |
| ☐ | ☒ | A statement on whether measurements were taken from distinct samples or whether the same sample was measured repeatedly |
| ☐ | ☒ | The statistical test(s) used AND whether they are one- or two-sided<br>*Only common tests should be described solely by name; describe more complex techniques in the Methods section.* |
| ☒ | ☐ | A description of all covariates tested |
| ☒ | ☐ | A description of any assumptions or corrections, such as tests of normality and adjustment for multiple comparisons |
| ☐ | ☒ | A full description of the statistical parameters including central tendency (e.g. means) or other basic estimates (e.g. regression coefficient) AND variation (e.g. standard deviation) or associated estimates of uncertainty (e.g. confidence intervals) |
| ☐ | ☒ | For null hypothesis testing, the test statistic (e.g. *F*, *t*, *r*) with confidence intervals, effect sizes, degrees of freedom and *P* value noted<br>*Give P values as exact values whenever suitable.* |
| ☒ | ☐ | For Bayesian analysis, information on the choice of priors and Markov chain Monte Carlo settings |
| ☒ | ☐ | For hierarchical and complex designs, identification of the appropriate level for tests and full reporting of outcomes |
| ☒ | ☐ | Estimates of effect sizes (e.g. Cohen's *d*, Pearson's *r*), indicating how they were calculated |

*Our web collection on statistics for biologists contains articles on many of the points above.*

## Software and code

Policy information about availability of computer code

| | |
|---|---|
| Data collection | Leica software was used to acquire images, MaxQuant v1.6.6 and Perseus 1.4.0.2 were used for mass spectrometry. |
| Data analysis | For RNA sequence analysis CutAdapt 1.5, R package RSEM 1.3.0 and R package pheatmap 1.0.8 were used. Gene enrichment analysis of complete pre ranked gene list was done using GSEA 4.0.1. GraphPad Prism 7 software, R3.3.1 or R 3.5.1 was used for statistical analysis. |

For manuscripts utilizing custom algorithms or software that are central to the research but not yet described in published literature, software must be made available to editors/reviewers. We strongly encourage code deposition in a community repository (e.g. GitHub). See the Nature Research guidelines for submitting code & software for further information.

## Data

Policy information about availability of data

All manuscripts must include a data availability statement. This statement should provide the following information, where applicable:

- Accession codes, unique identifiers, or web links for publicly available datasets
- A list of figures that have associated raw data
- A description of any restrictions on data availability

*Provide your data availability statement here.*

# Field-specific reporting

Please select the one below that is the best fit for your research. If you are not sure, read the appropriate sections before making your selection.

☒ Life sciences ☐ Behavioural & social sciences ☐ Ecological, evolutionary & environmental sciences

# Life sciences study design

All studies must disclose on these points even when the disclosure is negative.

| | |
|---|---|
| Sample size | No sample size calculations were done. We have included at least 3 animals for each age group and genotype. |
| Data exclusions | no data was excluded |
| Replication | All data presented here is reproduced (as shown in the data where n numbers are stated). There was no cases of datasets where our results were not reproduced. |
| Randomization | There was no randomization done, this was not possible |
| Blinding | Where possible the researcher was blinded to the genotype of the animal during analysis of the brains. Often this was not possible due to the clear phenotypic differences between genotypes. |

# Reporting for specific materials, systems and methods

We require information from authors about some types of materials, experimental systems and methods used in many studies. Here, indicate whether each material, system or method listed is relevant to your study. If you are not sure if a list item applies to your research, read the appropriate section before selecting a response.

## Materials & experimental systems

| n/a | Involved in the study | |
|---|---|---|
| ☐ | ☒ | Antibodies |
| ☒ | ☐ | Eukaryotic cell lines |
| ☒ | ☐ | Palaeontology |
| ☐ | ☒ | Animals and other organisms |
| ☐ | ☒ | Human research participants |
| ☒ | ☐ | Clinical data |

## Methods

| n/a | Involved in the study | |
|---|---|---|
| ☒ | ☐ | ChIP-seq |
| ☒ | ☐ | Flow cytometry |
| ☒ | ☐ | MRI-based neuroimaging |

## Antibodies

| | |
|---|---|
| Antibodies used | YAP1 (1:400, Cell Signalling #14074), HOPX (1:250, Proteintech #11419-1-AP), NF-kB p65 (1:800, Cell Signalling), AMOTL2 (1:100 GeneTex # CTX120712), ANKRD1 (1:100, Proteintech #11427-1-AP), AXL (1:100, R&D Systems #AF854), C3 (1:100, Abcam #ab11862), Cre (1:500, Covance #PRB-106P), Ctip2 (1:500, Abcam #ab18465), GFAP (mouse 1:500, Sigma #G6171 and chicken 1:1000, Abcam #ab134436), HOPX (1:100, Proteintech #11419-1-AP), Ki67 (1:100/1:300, BD #550609), MUC1 (1:500, Abcam #ab45167), Nestin (1:100, Millipore #MAB353), NeuN (1:100, Millipore #MAB377) and YAP1 (rabbit 1:100, CST #14074 and mouse 1:100, Santa Cruz Biotechnology #sc-101199)CK18 (1:3000, ThermoFisher #PA5-14263; Tris-EDTA pH9), GFAP (1:750, DAKO #z0334), HOPX (1:250, Proteintech #11419-1-AP), Ki67 (1:350, Abcam #ab16667), MUC1 (1:500, Abcam #ab15481), Nestin (1:600, BD Biosciences #611659), Rela/ NF-kB p65 (1:800, Cell Signalling #8242), (NeuN (1:600, Chemicon #MAB377), Vimentin (1:600, Abcam #ab92547), YAP1 (1:400, CST #14074) |
| Validation | We have validated the YAP1 (CST 14074) in this study, by showing absence of staining in the center of the tumour in conditional knockout mice, where YAP is deleted and tumour progression is manifested by TAZ. For all other antibodies we rely on manufacturer's descriptions of the 1) endogenous protein bands observed in Western blots from various tissues or cell lines, these bands should be at the correct molecular weight 2) stainings presented in manufacturer's site. Where possible we used rabbit monoclonal antibodies, which have very low cross reactivity in westerns and stainings. Where possible we combined Western blots and stainings to confirm our findings. |

## Animals and other organisms

Policy information about studies involving animals; ARRIVE guidelines recommended for reporting animal research

| | |
|---|---|
| Laboratory animals | Nex-Cre (Neurod6tm1(cre)Kan, MGI:2668659), Lats1f/f Lats2f/f Lats1(tm1.1Jfm) MGI:5568586 and Lats2(tm1.1Jfm) MGI:5568589 , Ai14 (Gt(ROSA)26Sortm14(CAG-tdtomato)Hze), YAP1f/f (Yap1tm1c(KOMP)Mbp, MGI:5603606), TAZf/f (Wwtr1tm1c(EUCOMM)Wtsi, MGI:5603602), animals are crossed to C57Bl6 background. The age information is listed in detail in the manuscript. The gender information is not recorded. |
| Wild animals | n/a |
| Field-collected samples | n/a |

| | |
|---|---|
| Ethics oversight | Home Office Project Licence for Ultanir laboratory is used. We followed Animal (Scientific procedures) Act 1986 of the United Kingdom and protocols were approved by institutional (Francis Crick Institute) ethical reviews. |

Note that full information on the approval of the study protocol must also be provided in the manuscript.

## Human research participants

Policy information about studies involving human research participants

| | |
|---|---|
| Population characteristics | n/a |
| Recruitment | n/a |
| Ethics oversight | Written consent was given by the patient or by legal representatives (Felipe Andreiuolo), University of Bonn Medical Center. |

Note that full information on the approval of the study protocol must also be provided in the manuscript.
