# Supplementary figures and images for: YAP1/TAZ drives ependymoma-like tumour formation in mice
Source: Nat Commun. 2020 May 13;11:2380. doi: 10.1038/s41467-020-16167-y (PMC7220953; doi:10.1038/s41467-020-16167-y)

Full Western blot scans Figure 7a

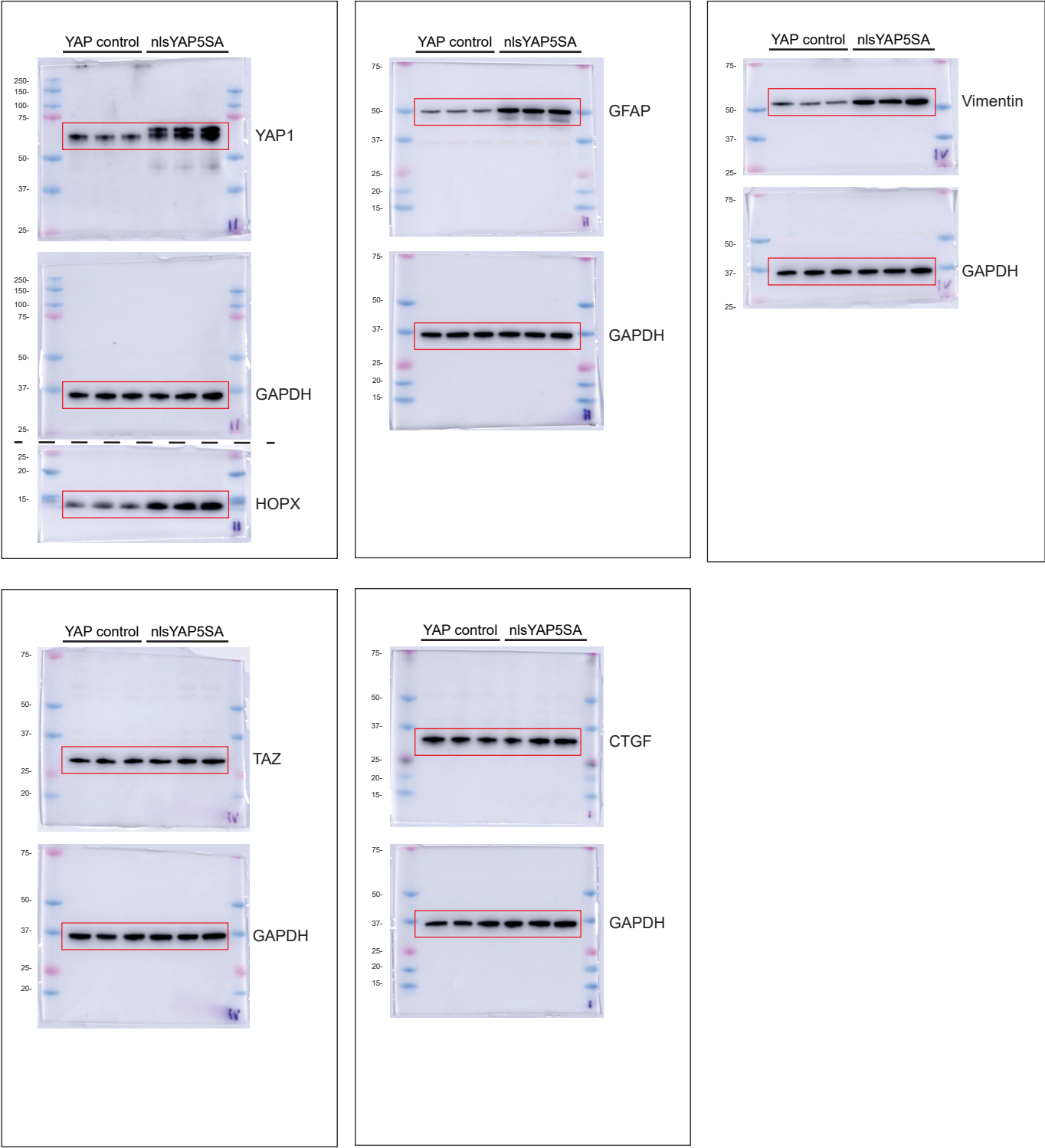

Supplement: Supplementary file 8 — Source Data [file 41467_2020_16167_MOESM8_ESM.pdf]
