# Supplementary material for: YAP1/TAZ drives ependymoma-like tumour formation in mice
Source: Nat Commun. 2020 May 13;11:2380. doi: 10.1038/s41467-020-16167-y (PMC7220953; doi:10.1038/s41467-020-16167-y)
Supplement: Supplementary file 1 — Supplementary Information [file 41467_2020_16167_MOESM1_ESM.pdf]

YAP1/TAZ drives ependymoma-like tumour formation in mice

Eder et al.,

Supplementary Information

Supplementary Figure 1

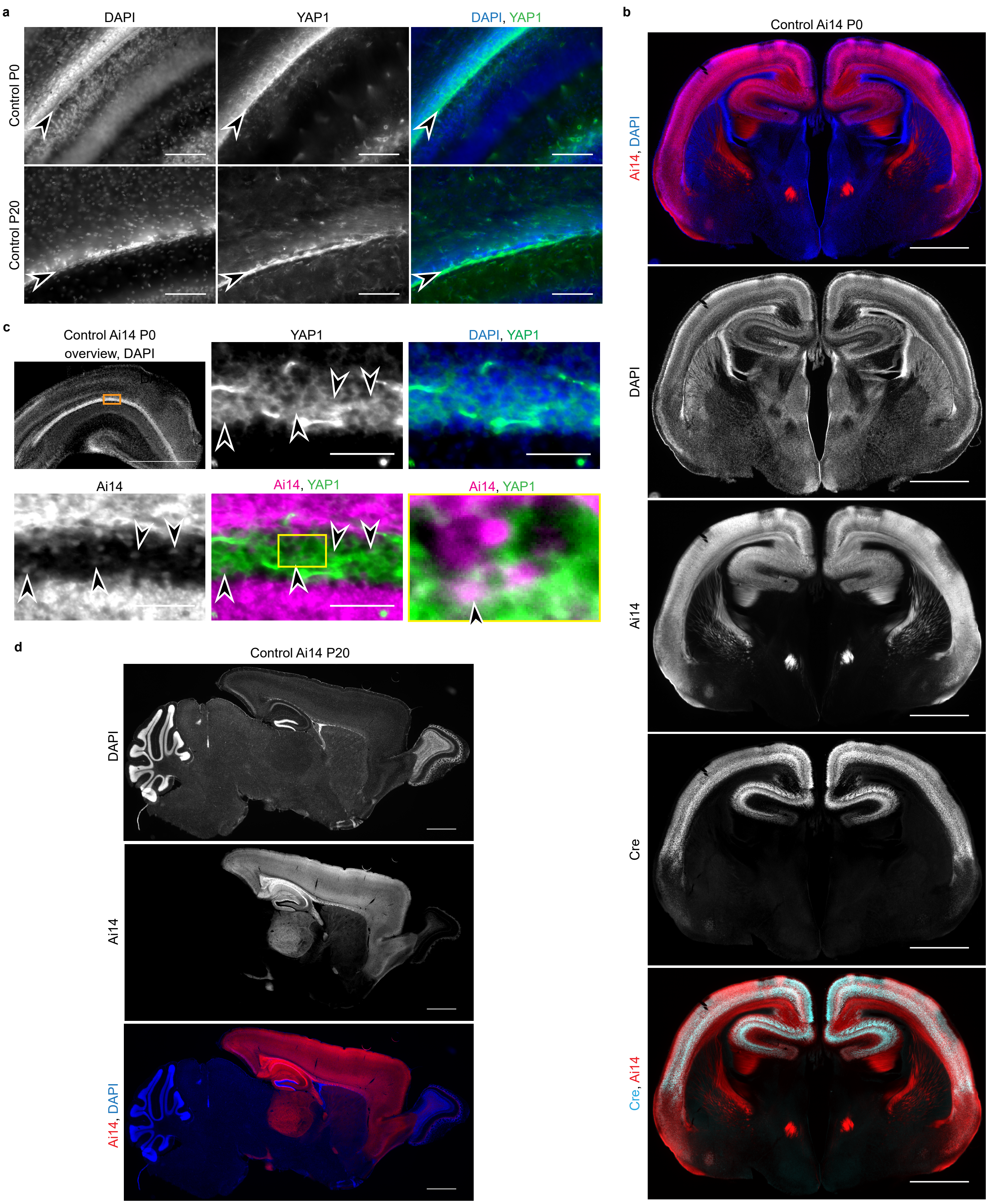

Supplementary Figure 1: YAP expression in ependymal layer of the murine brain and Cre-mediated recombination in the mouse brain confirmed by Ai14 reporter.

a. Immunofluorescence staining of YAP1 in vibratome sections of a P0 and a P20 control brain. Arrowheads pointing at YAP1 positive ependymal layer. Scale bars = 100 µm. **b.** Coronal vibratome section of a P0 NEX-Cre/+ Ai14 brain. Cre and reporter expression detected in the cerebral cortex and hippocampus in addition axon tracks are visible with the reporter. Scale bar = 1 mm **c.** Higher magnification of a P0 ependymal layer showing the presence of YAP1 and Ai14 positive cells. Scale bars, overview = 1 mm, detail = 50 µm. **d.** Sagittal vibratome section of a P20 NEX-Cre/+ Ai14 brain. Reporter expression detected in the cerebral cortex and hippocampus including the axon tracks of the targeted neurons. Scale bars = 1 mm.

Supplementary Figure 2

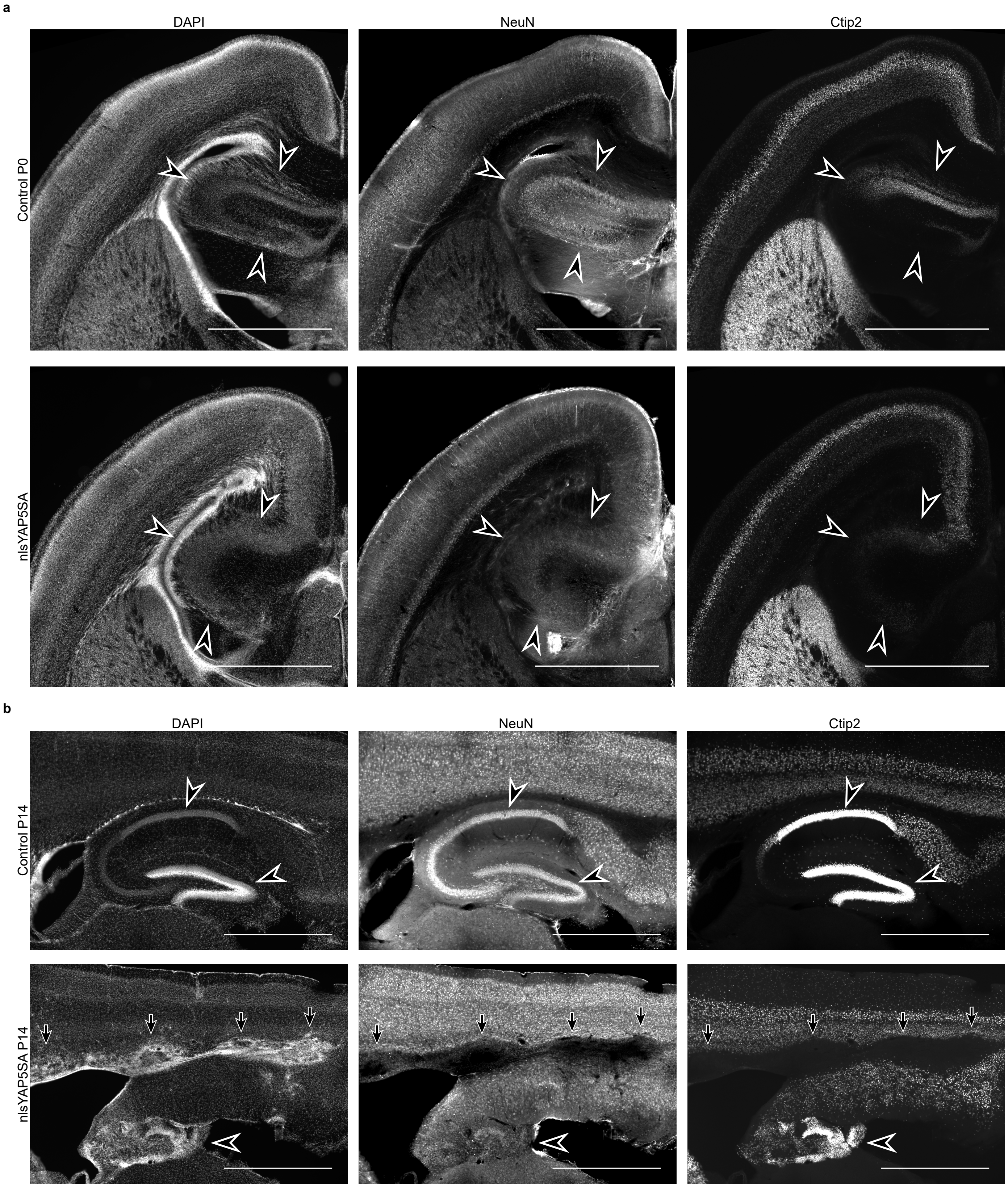

Supplementary Figure 2: Impaired hippocampal development in NEX-Cre nlsYAP5SA animals.
a. Immunofluorescence staining of coronal vibratome sections of P0 animals. NeuN and Ctip2 reveal a reduction of positively stained cells in the nlsYAP5SA animal compared to the control littermate. Arrowheads indicating hippocampus. Scale bars = 1 mm. **b.** Immunofluorescence staining of sagittal vibratome sections of P14 animals. Distorted ependymal layer is absent for NeuN and Ctip2 staining (arrows). Hippocampus of the nlsYAP5SA animal is heavily affected and lacks any clear CA1-CA3-DG structure which can be seen in the control littermate (arrowheads). Scale bars = 1 mm.

Supplementary Figure 3

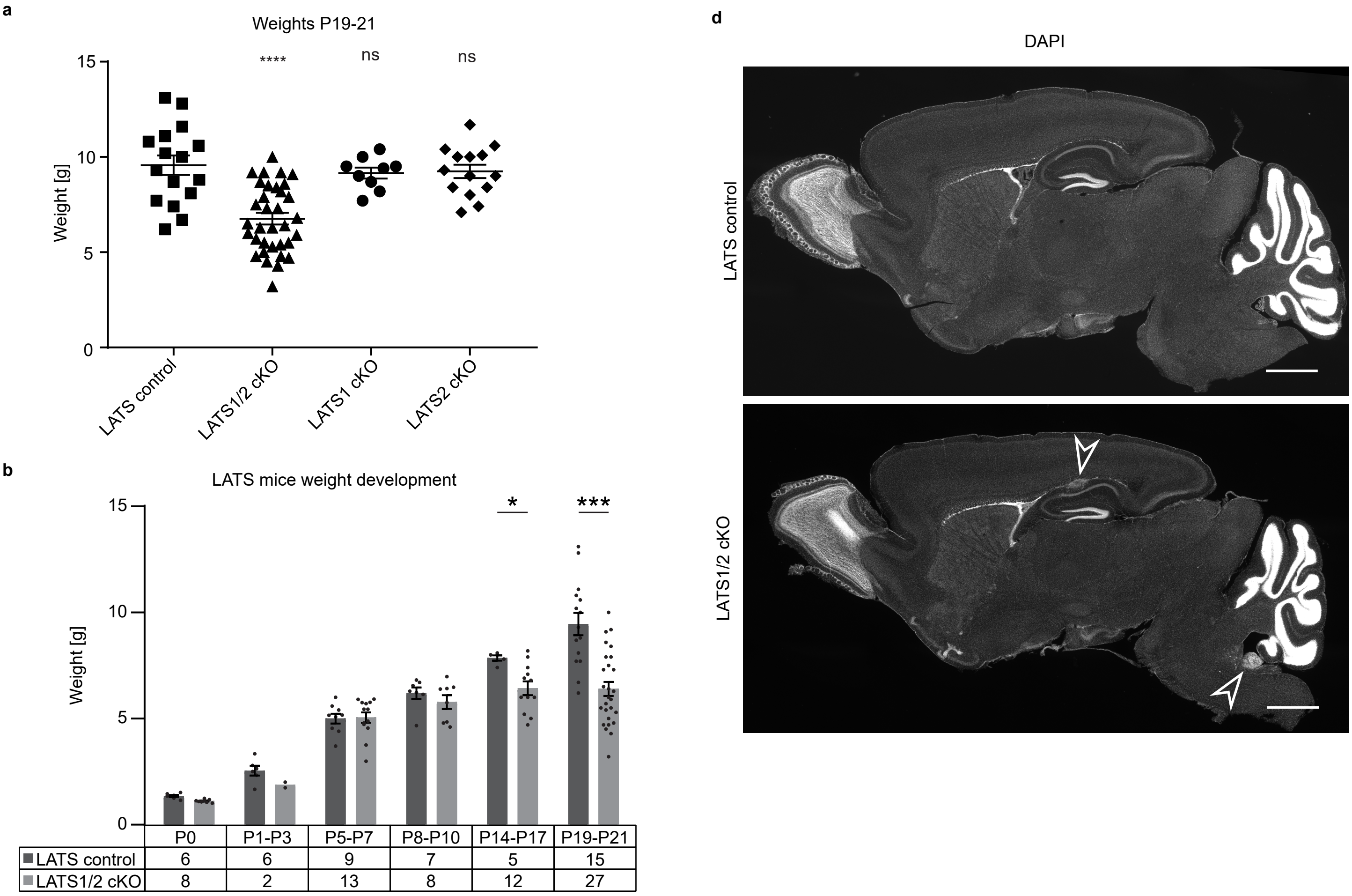

c

Age range	P0-P2	P5-P7	P8-P10	P19-P21
Sectioned brains	3	3	3	15
% Tumours detected	0%	67%	100%	100%

Supplementary Figure 3: Tumour occurrence in the LATS1/2 cKO model

a. LATS control, LATS1/2 cKO, LATS1 cKO ($NEX^{Cre/+}$ $Lats1^{f/f}$ $Lats2^{+/+}$) and LATS2 cKO ($NEX^{Cre/+}$ $Lats1^{+/+}$ $Lats2^{f/f}$) were weighted at the age of P19-P21 revealing a significant difference between the LATS control and the conditional double knockout animals (padj < 0.0001, two-sided Dunnett`s multiple comparisons test), but not the single knockout. N = 16 LATS1/2 control, 34 LATS1/2 cKO, 9 LATS1 cKO, 14 LATS2 cKO. Error bars show SEM. **b.** Weights of animals at multiple ages comparing LATS control and LATS1/2 cKO animals. Significant difference found at P14-17 (p = 0.04, two-sided Student`s t-test) and at P19-P21 (p=0.00016, two sided Student t-test). Number of weight animals per age group indicated below divided by genotype. Each dot represents one weight animal. Error bars show SEM. **c.** Number of sectioned animals per age group and percentage of how many had visible tumours identified. **d.** Sagittal vibratome section of LATS control and LATS1/2 cKO animals. In addition to the tumour at the ependymal layer, in a subset of LATS1/2 cKO animals also a tumour located at the cerebellum can be found, arrow heads pointing at tumours. Scale bar = 1 mm.

Supplementary Figure 4

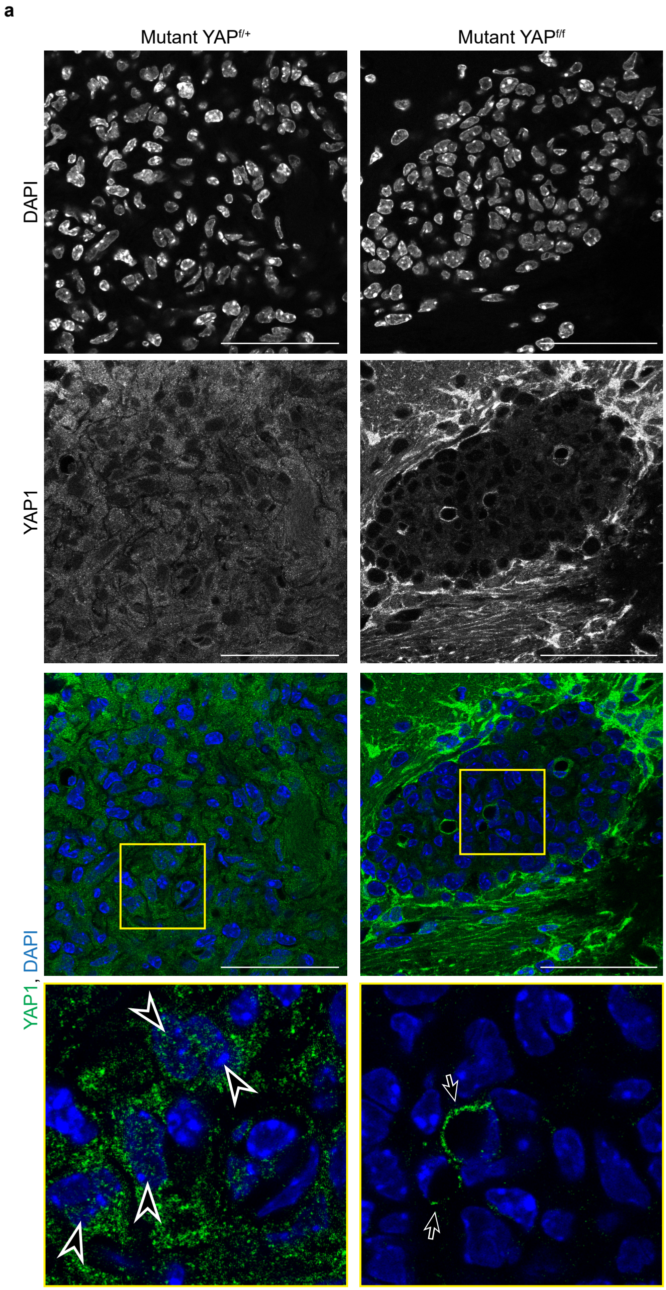

Supplementary Figure 4: Specificity of anti-YAP1 antibody immunostaining

a. YAP1 staining is shown in brain tumour sections from Lats1$^{f/f}$ Lats2$^{f/f}$ YAP1$^{f/+}$ TAZ$^{+/+}$ (left, Mutant YAP$^{f/+}$) and Lats1$^{f/f}$ Lats2$^{f/f}$ YAP1$^{f/f}$ TAZ$^{+/+}$ (right, Mutant Yap$^{f/f}$) mice. In Mutant YAP$^{f/+}$ mice YAP1 staining is present in the tumour mass and inside the nucleus in subset of cells (arrowheads). In mice tumours where YAP1 is deleted (Mutant YAP$^{f/f}$) there is no YAP1 staining in tumour centre, staining is only observed in blood vessels, where YAP1 is not deleted (arrow). Scale bars = 50 µm.

Supplementary Figure 5

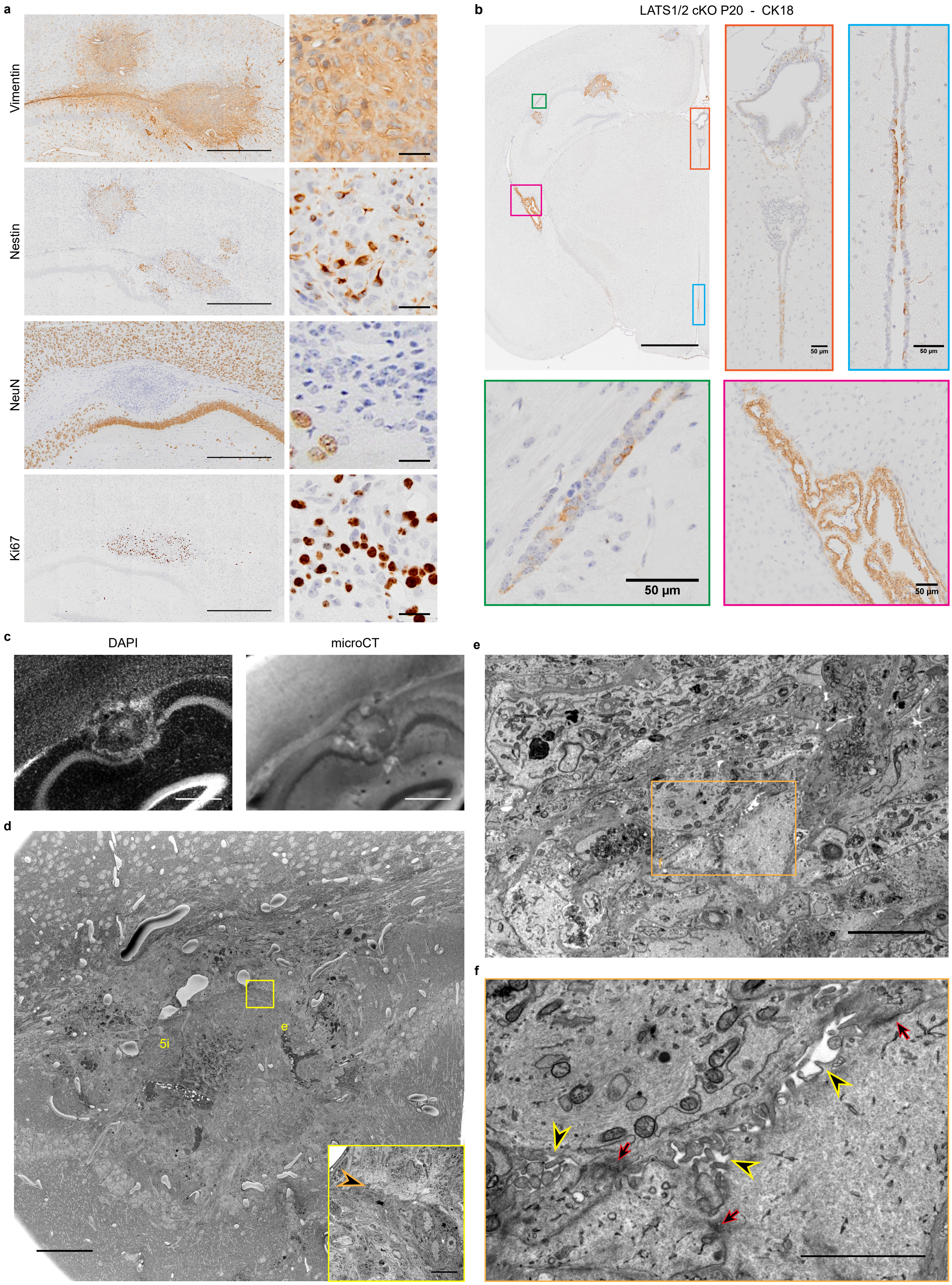

Supplementary Figure 5: LATS1/2 cKO tumour immunohistochemistry and ultrastructure

a. IHC stained P20 LATS1/2 cKO brains with multiple markers. Tumours show positive staining for Vimentin, Nestin and Ki67. The tumour is negative for NeuN. Scale bars, overview = 500 µm, detail = 20 µm. **b.** IHC stained P20 LATS1/2 cKO brain with Cytokeratin 18 demonstrating positive staining in various ependymal layer across the brain. Scale bars, overview = 1 mm, details = 50 µm. **c.** DAPI stained 50 µm vibratome section and 3D reconstruction of the micro-CT images of the consecutive 100 µm section used for SBF SEM and TEM. Tumour localised in the white frame on the DAPI image. Scale bar = 500 µm. **d.** Last low resolution SBF SEM overview image of the tumour acquired before manual ultrathin sectioning for TEM. The letters indicate the regions of interest further imaged by TEM, shown in e and Fig.5i. The inset, enlargement of the white square, shows the border of the tumour. Scale bars, overview = 100 µm, detail = 10 µm. **e.** TEM image (2550x) of one region of interest within the tumour. Scale bar = 5 µm. **f.** TEM image (8200x) of the orange inset indicated in e showing features of ependymoma in a murine tumour. Scale bar = 2.5 µm. Yellow arrow heads = microvilli, red arrows = tight junctions.

Supplementary Figure 6

a

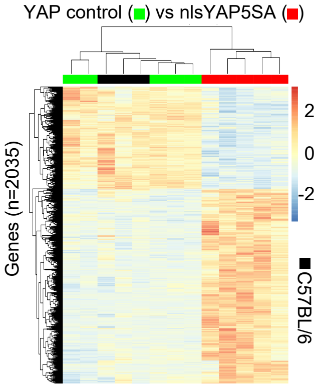

f

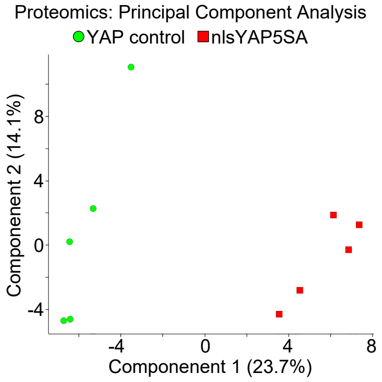

g

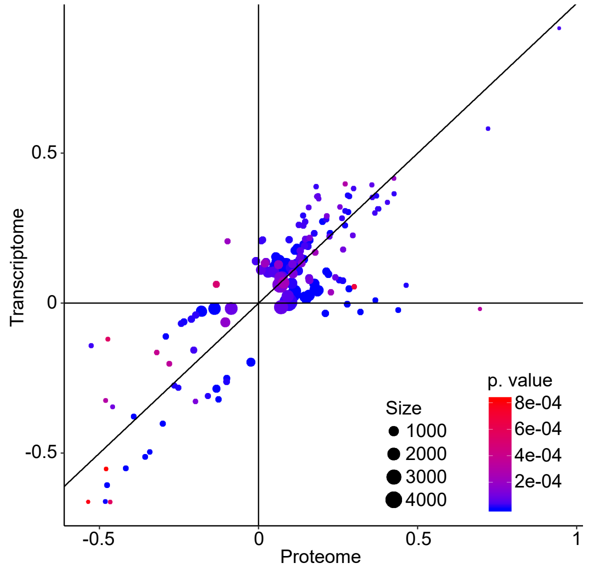

b

KEGG Name (negative Top10)	NES	Size
Parkinsons Disease	-7.169	107
Oxidative Phosphorylation	-7.133	109
Huntingtons Disease	-5.818	154
Alzheimers Disease	-5.724	145
Ribosome	-4.540	82
Citrate Cycle Tca Cycle	-3.353	28
Cardiac Muscle Contraction	-2.905	74
Steroid Biosynthesis	-2.820	16
Axon Guidance	-2.704	127
Proteasome	-2.672	43

h

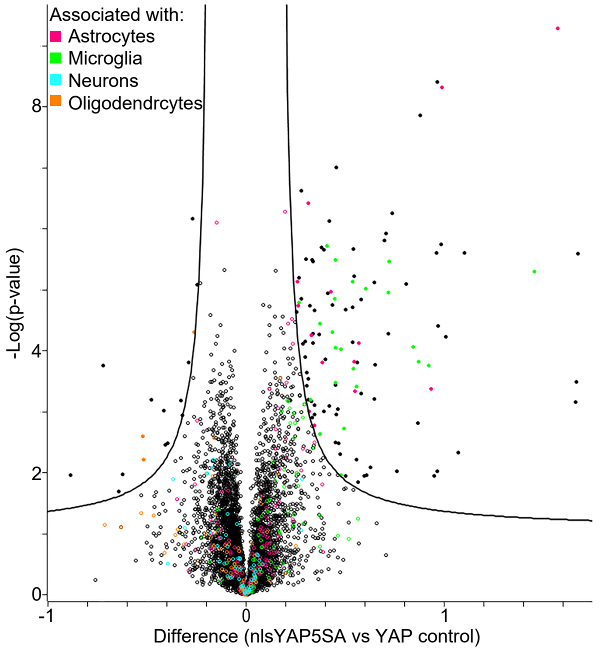

c

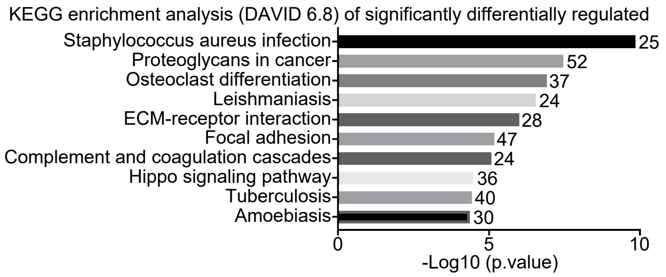

d

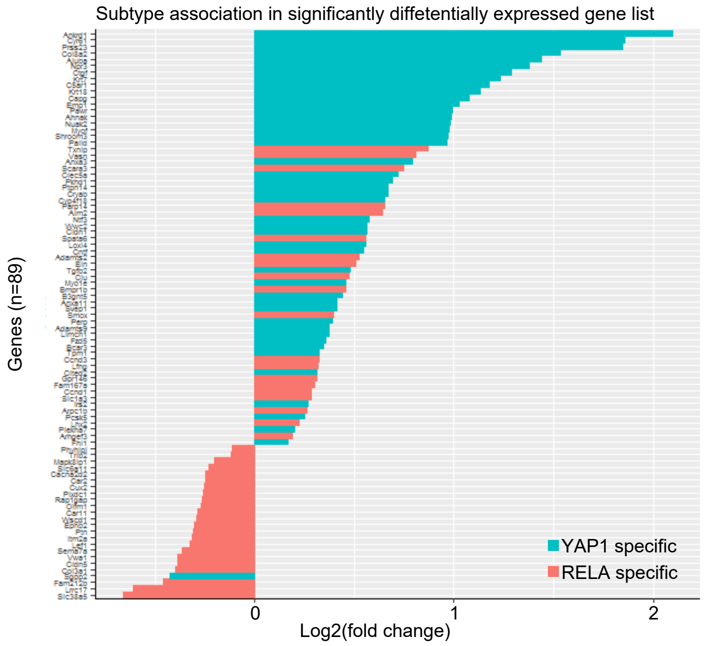

i

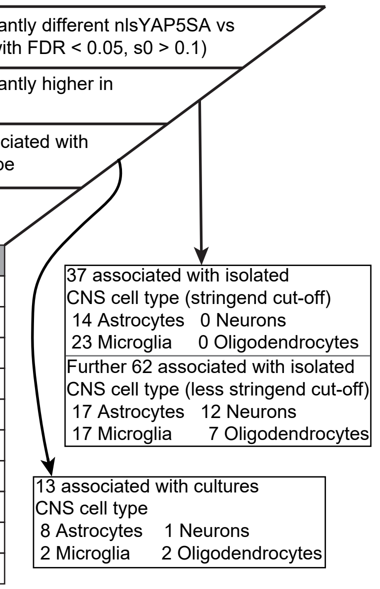

	Gene	Uniprot
e.c.	Eln	P54320
	Hopx	Q8R1H0
	Ifitm2	Q99J93
e.c.	Serpinf1	P97298
e.c.	Kng1	O08677
e.c.	Hba	P01942
	Tpm1	E9Q452
e.c.	Pzp	Q61838
e.c.	Ahsg	P29699
e.c.	Cfb	P04186

e

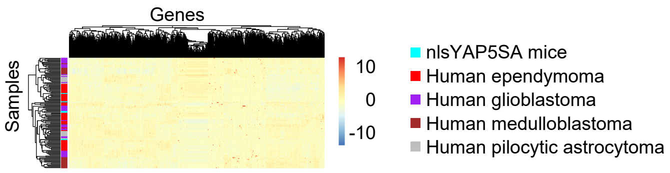

Supplementary Figure 6: Transcriptional and proteomics landscape of nlsYAP5SA in comparison to control.

a-d. Gene expression analysis for nlsYAP5SA versus control. Five animals per genotype (control = $NEX^{+/+}$ $YAP1^{nlsYAP5SA/+}$; nlsYAP5SA = $NEX^{Cre/+}$ $YAP1^{nlsYAP5SA/+}$) were analysed utilising the Illumina HiSeq 4000. **a.** Heat map of genes with significantly differently expression between control and nlsYAP5SA animals showing additional three C57BL/6 animals which were processed at the same time to control for any leakage of nlsYAP5SA in our controls to the background. Rows represent genes and columns show individual animals. Legend indicates fold change. **b.** Gene enrichment analysis (GSEA) of complete gene list from RNASeq with gene sets derived from the KEGG pathway database. Top ten annotated pathways with the lowest normalised enrichment score (NES) are shown with number of genes linked to the indicated pathway (Size). **c.** Pathway enrichment analysis with DAVID 6.8 using significant differentially expressed genes list annotating with the KEGG defined gene set. Top ten enriched pathways ranked by p-value are shown with the number of associated genes within the supplied dataset. **d.** mRNA expression that is significantly changed in nlsYAP5SA mice is shown. For YAP1 specific genes: 1 is reduced and 45 are increased out of 46 mRNAs associated with YAP1 that are significantly changed in nlsYAP5SA mouse model. For RELA specific genes 20 are increased and 23 are reduced out of 43 significantly changed genes in nlsYAP5SA model. **e.** Attempted cluster gene expression analysis comparing nlsYAP5SA with previously published microarray data from pediatric human brain cancers [58]. No clear clustering and inconclusive result, predominantly due to considerably technical caveats on comparing Affymetrix microarray data with next generation sequencing. **f.** Principal component analysis (PCA) performed on acquired full proteome dataset of nlsYAP5SA versus control. Five animals per genotype utilising TMT 10plex. Clear separation between the two experimental groups can be observed. **g.** 2D annotation enrichment based on measured protein and mRNA of genes present in proteome (x-axis) and transcriptome (y-axis) datasets (matched by gene name). Gene ontology (GO – BP, MF, CC) and KEGG annotation done in Perseus v1.4.0.2. Each dot represents one annotation, the size indicates the associated number of genes and the colour the corresponding p-value. **h.** Volcano plot of protein expression comparing nlsYAP5SA against control. Proteins associated with astrocytes (purple), microglia (green), neurons (pink) and oligodendrocytes (blue-green) are displayed. Cell type association was generated by integrating data published in Sharma *et al* [61]. In brief, association was defined by expression value being a minimum of 3 in one of the four isolated cell types (astrocytes, microglia, neurons and oligodendrocytes) and with a value of at least double in one compared to any of the other cell types. Each dot represents one protein. X-axis has been locked to [-1,1.75] excluding the display of one protein, eliminating Eif2s3y, a Y-linked protein, enriched due to the mixed gender of experimental animals. **i.** Filtering workflow used for proteome dataset by implementing previous published CNS cell type specific expression data from Sharma *et al* [61]. First, significance within proteome comparison of five mutant vs five control was determined by welch-t-test and set $FDR<0.05$ and $s0>0.1$. Of the 129 significantly differentially expressed proteins 113 were upregulated of which 99 were detected in isolated CNS cells in the Sharma et al 2015 full proteome dataset. The remaining 23 proteins were filtered against the cultured CNS cells date set of Sharma et al 2015. The majority of the lasting 10 proteins are described to be found secreted extracellular proteins, potentially explaining their lack of detection in the cell type specific dataset, leaving *Hopx*, *Ifirm2* and *Tmp1*.

Supplementary Figure 7

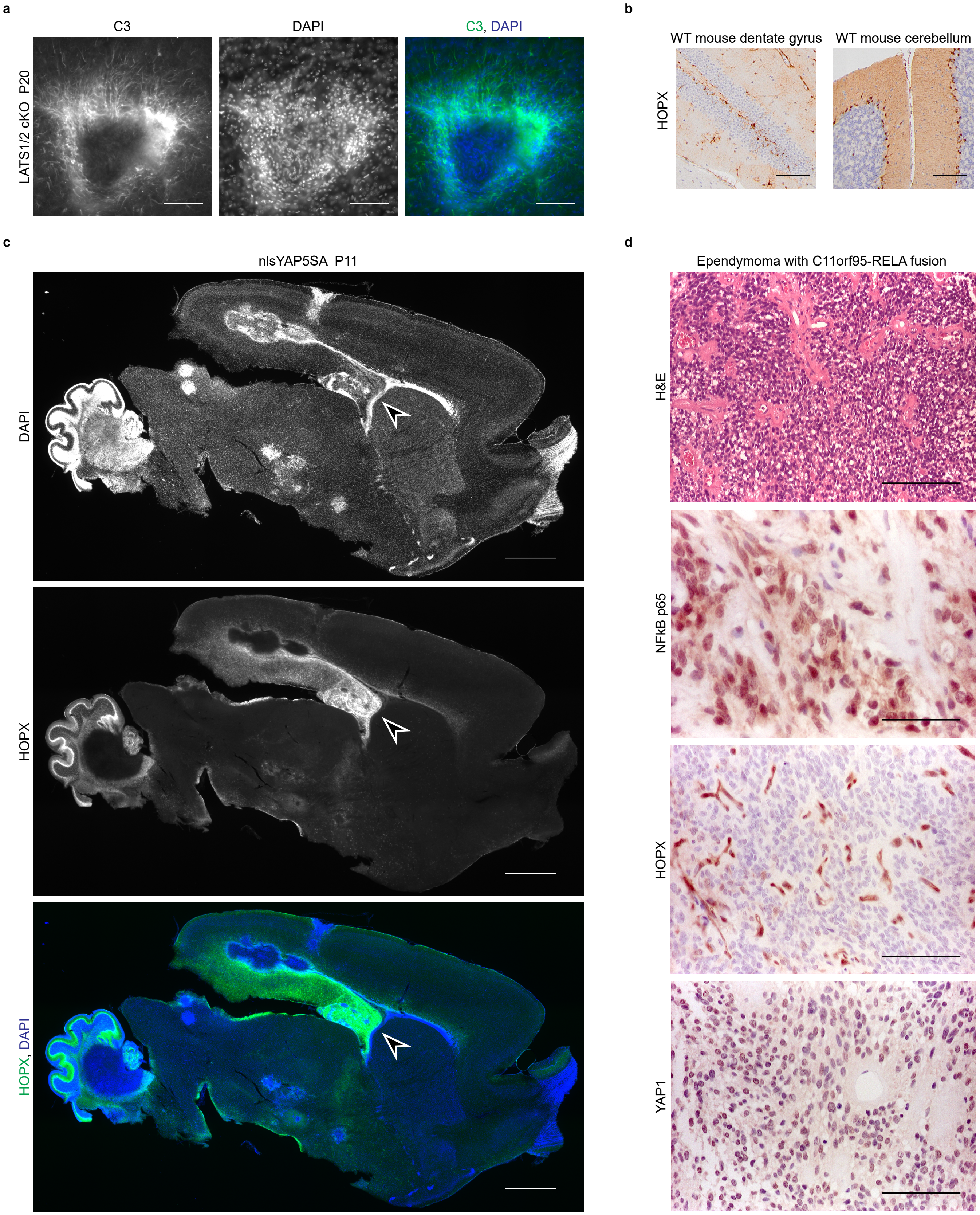

Supplementary Figure 7: Characterization of marker expression in mouse models and human ependymomaa. Immunofluorescence staining of C3 in coronal vibratome sections of P20 LATS12 cKO animal showing strong tumour boarder staining and reduced positive staining in centre. Scale bar = 100 µm. **b.** HOPX antibody positive control stained in parallel to human patient samples in Fig. 7e showing expected staining in dentate gyrus and cerebellum of adult wild type (WT) mouse. Scale bars = 100 µm. **c.** Immunofluorescence staining of HOPX in sagittal vibratome sections of P11 nlsYAP5SA animal showing strong staining in deformed hippocampal formation and tumour boarder regions. Scale bar = 1 mm. **d.** This case of ependymoma with C11orf95-RELA fusion shows densely cellular sheets of tumour cells with hyperchromatic nucleus; perivascular pseudorosettes are present (H&E, scale bar = 200 µm); tumour cells demonstrate nuclear and cytoplasmic p65 expression (immunoperoxidase, scale bar = 50 µm); HOPX in only expressed in endothelial cells while it is negative in tumour cells (immunoperoxidase, scale bar = 100 µm); There is no YAP1 expression in tumour cells (immunoperoxidase, scale bar = 100 µm).
